# Classification of Diabetes Using Feature Selection and Hybrid Al-Biruni Earth Radius and Dipper Throated Optimization

**DOI:** 10.3390/diagnostics13122038

**Published:** 2023-06-12

**Authors:** Amel Ali Alhussan, Abdelaziz A. Abdelhamid, S. K. Towfek, Abdelhameed Ibrahim, Marwa M. Eid, Doaa Sami Khafaga, Mohamed S. Saraya

**Affiliations:** 1 Department of Computer Sciences, College of Computer and Information Sciences, Princess Nourah bint Abdulrahman University, P.O. Box 84428, Riyadh 11671, Saudi Arabia; aaalhussan@pnu.edu.sa; 2 Department of Computer Science, College of Computing and Information Technology, Shaqra University, Shaqra 11961, Saudi Arabia; 3 Department of Computer Science, Faculty of Computer and Information Sciences, Ain Shams University, Cairo 11566, Egypt; 4 Computer Science and Intelligent Systems Research Center, Blacksburg, VA 24060, USA; sktowfek@jcsis.org; 5 Department of Communications and Electronics, Delta Higher Institute of Engineering and Technology, Mansoura 35111, Egypt; 6 Computer Engineering and Control Systems Department, Faculty of Engineering, Mansoura University, Mansoura 35516, Egypt; abdelhameedibrahim79@gmail.com (A.I.); mohamedsabry83@mans.edu.eg (M.S.S.); 7 Faculty of Artificial Intelligence, Delta University for Science and Technology, Mansoura 11152, Egypt; mmm@ieee.org

**Keywords:** diabetes, machine learning, feature selection, Al-Biruni earth radius optimization, dipper throated optimization, random forest

## Abstract

Introduction: In public health, machine learning algorithms have been used to predict or diagnose chronic epidemiological disorders such as diabetes mellitus, which has reached epidemic proportions due to its widespread occurrence around the world. Diabetes is just one of several diseases for which machine learning techniques can be used in the diagnosis, prognosis, and assessment procedures. Methodology: In this paper, we propose a new approach for boosting the classification of diabetes based on a new metaheuristic optimization algorithm. The proposed approach proposes a new feature selection algorithm based on a dynamic Al-Biruni earth radius and dipper-throated optimization algorithm (DBERDTO). The selected features are then classified using a random forest classifier with its parameters optimized using the proposed DBERDTO. Results: The proposed methodology is evaluated and compared with recent optimization methods and machine learning models to prove its efficiency and superiority. The overall accuracy of diabetes classification achieved by the proposed approach is 98.6%. On the other hand, statistical tests have been conducted to assess the significance and the statistical difference of the proposed approach based on the analysis of variance (ANOVA) and Wilcoxon signed-rank tests. Conclusions: The results of these tests confirmed the superiority of the proposed approach compared to the other classification and optimization methods.

## 1. Introduction

Hyperglycemia due to abnormalities in insulin secretion, insulin action, or both characterize the metabolic condition known as diabetes mellitus (DM). Long-term damage, dysfunction, and failure of multiple organs, including the heart, eyes, kidneys, blood vessels, and nerves, can be attributed to the chronic hyperglycemia that is associated with DM [1]. Diabetes mellitus (DM) is divided into three subtypes based on its etiology and clinical presentation: type 1 diabetes (T1DM), type 2 diabetes (T2DM), and gestational diabetes. In type 1 diabetes, beta cells in the pancreas are destroyed, typically as a result of a cellular-mediated autoimmune response, leading to an utter lack of insulin. Type 2 diabetes is brought on by insulin resistance and a little insulin shortage. Gestational diabetes, often known as pregnancy-onset diabetes, is characterized by glucose intolerance of varied degrees [2]. The vast majority of diabetes cases are type 2 diabetes. Although type 2 diabetes is more common in people over 40, it can also affect those of any age. The symptoms of this type may not manifest for years, and many individuals are diagnosed by chance when they seek care for unrelated issues. Patients with T2DM are not insulin-dependent, but they may require insulin therapy to manage hyperglycemia if it cannot be achieved via diet alone or with oral hypoglycemic medications [3].

The causes of type 2 diabetes are varied and convoluted. Many factors increase or decrease the likelihood of contracting the disease, although not all of them are direct causes. These factors may be hereditary, demographic (such as age), or behavioral (food, smoking, obesity, and lack of exercise). Behavioral risk factors are sometimes referred to as “modifiable” risk factors [4] since they can be altered or improved upon. More than 460 million individuals worldwide were diagnosed with diabetes in 2019, and that number is anticipated to rise to 578 million by 2030 and 700 million by 2045, as reported by the International Diabetes Federation (IDF) [5]. The prevalence of type 2 diabetes mellitus (T2DM) continues to rise, making it one of the most concerning NCDs.

To predict the future incidence and global prevalence of diabetes, numerous studies over the past two decades have used various data sets and analytical methodologies [6,7]. Accurate forecasts of the future burden of diabetes are vital for health policy planning and establishing the costs of managing the condition [8,9]. Disease prediction and diagnosis for pandemic chronic diseases such as diabetes are two areas where machine learning algorithms have lately seen extensive application in public health. Machine learning techniques examine historical patterns in data to foretell what will happen in the future. Algorithms in machine learning provide data modeling, analysis, and visualization mention [10,11]. Several machine learning techniques have been utilized in diabetes modeling studies, including support vector machines (SVMs), artificial neural networks (ANNs), k-nearest neighbor (KNN), and decision trees (DTs) [12,13]. Despite the importance of studying diabetes prevalence trends and predicting future burdens using risk factors in specific populations, little work has been done to adopt machine learning classification methods.

Feature selection is essential in analyzing healthcare datasets, such as diabetes-related ones. The goal is to find the small subset of features that makes the most significant impact on a task of classification or prediction. The problem is high-dimensional datasets, which frequently have extraneous or redundant features that can cause overfitting and lower classification accuracy. Researchers have looked into using metaheuristic optimization techniques for feature selection in diabetic datasets to address this problem. On the other hand, metaheuristic optimization algorithms provide effective means of exploring and ultimately settling on subsets of features that can be used to improve machine learning performance. These algorithms take their cues from real-world occurrences or problem-solving techniques, and they’re built to efficiently probe the range of possible solutions. These algorithms aim to make diabetes categorization models more accurate, less dimensional, and easier to read [14,15].

Feature selection is challenging in machine learning due to several inherent difficulties. One of the primary challenges is dealing with high-dimensional datasets that contain a large number of features. High dimensionality often leads to increased computational complexity, reduced model interpretability, and the risk of overfitting. Additionally, datasets may include irrelevant or redundant features that can adversely affect the performance of machine-learning models. Metaheuristic optimization methods offer promising solutions to address these challenges. By leveraging these the exploration and exploitation capabilities of these algorithms, researchers can enhance the feature selection accuracy, reduce dimensionality, and optimize the performance of machine learning models for tasks such as diabetes classification [16,17].

In this paper, a new optimization algorithm is proposed for feature selection and optimization of the parameters of the random forest classifier. The proposed optimization algorithm is a dynamic hybrid of the Al-Biruni Earth Radius and Dipper-Throated Optimization algorithm and is denoted by (DBERDTO). The main advantage of this algorithm is the improved exploration and exploitation of the search space while performing the optimization process. This advantage appears in the promising classification results, which are superior to those of the other competing methods. The following is a summary of the novelty of this work:

- A new binary optimization algorithm (bDBERDTO) is proposed for feature selection to select the most significant set of features that can improve the classification results;
- A new optimization algorithm (DBERDTO) is proposed to optimize the parameters of the random forest classifier to boost the classification accuracy;
- A comparison with the other feature selection methods is performed to prove the superiority of the proposed feature selection method;
- A comparison with the other classification models is performed to show the effectiveness of the proposed optimization method and the proposed approach to optimizing the parameters of the random forest classifier;
- A statistical analysis is performed to show the stability and statistical difference between the proposed approach and the other competing approaches;
- The ANOVA and Wilcoxon signed rank tests are performed, and the results are analyzed to show the effectiveness of the proposed methodology;
- The proposed approach is evaluated in terms of four diabetes datasets available on Kaggle to prove its effectiveness and generalization.

The paper proceeds as follows. There is a literature review in Section 2. Section 3 discusses the proposed methodology. The experimental approach is analyzed in Section 4. In Section 5, the outcomes are concluded and the future perspectives are highlighted.

## 2. Literature Review

Machine learning algorithms have found extensive application in public health, particularly in disease prediction and diagnosis for chronic epidemic conditions such as diabetes. Various machine learning methods, such as support vector machines, artificial neural networks, k-nearest neighbors, and decision tree models, have all been used in diabetes models that have been published. Various applications have found success with these models, including the early detection of diabetes and the modeling of its consequences. To compare the suggested classifiers’ accuracy with existing ones, this section includes the commonly used machine learning techniques and their respective accuracy rates. Table 1 provides a summary of all the research that is mentioned in this section. In a 2019 study, Ref. [18] compared the performance of various classification models for diabetes prediction using a variety of machine learning techniques (including SVM, C4.5 decision tree, naive Bayes, and KNN) and evaluation metrics (accuracy, recall, and precision). Medical Centre Chittagong (MCC) in Bangladesh served as the source for the diabetes data used in the study. There are 200 patients in the dataset, and they all have unique features, including age, sex, weight, blood pressure, and other potential health issues. Study findings showed that the C4.5 decision tree model performed the best, with an accuracy of 73%. The authors of Ref. [19], another study published in 2018, set out to assess how well categorization algorithms might foretell cases of diabetes. A total of 768 samples were used in this analysis, all of which came from the PIMA Indian data repository. Seventy percent (*n* = 583) of the data was used for training, whereas thirty percent (*n* = 231) was used for testing. Logistic regression (LR), k-nearest neighbor (KNN), support vector machine (SVM), gradient boost, decision tree (DT), boosted DT, boosted MLP, random forest (RF), and Gaussian naive Bayes (GNB) were the eight machine learning models tested in this work. According to the findings, LR’s accuracy was the highest at 79.54%.

The authors in Ref. [20] built an ANN model with varying numbers of hidden network neurons (from 5 to 50). Female diabetes was predicted using information from the National Institute of Diabetes and Digestive and Kidney Diseases. Using the Pima Indian Diabetes dataset for validation and the assessment measures of accuracy and mean squared error (MSE), it was shown that the ANN model trained with 8 features had a 93% accuracy. Using AdaBoost, k-NN, decision trees, XG Boost, naive Bayes, and random forest, the authors of Ref. [21] conducted a study in 2020 to diagnose and forecast the onset of diabetes. A total of 768 female patients were used to train the models, 268 of whom had diabetes (positive) and 500 did not (negative). This analysis considered eight factors: blood sugar, insulin, pregnancy, blood pressure, triceps strength, body mass index, family history, and age. Feature selection, data normalization, outlier rejection, mean substitution for missing values, and k-fold cross-validation (five-fold cross-validation) were all part of the data preparation process [22]. An ensemble method was also employed to improve performance with numerous classifiers further. The predictive power of ensemble methods can be increased by combining the results from multiple models. When combined, AdaBoost and XGBoost produced the highest-quality results. The area under the curve (AUC) was used as the effectiveness measure. An AUC of 0.95 was achieved, making their study the most accurate.

In Ref. [23], the authors from several institutions used the PIDD dataset to create a number of machine-learning models to identify whether or not a given female patient has diabetes. The mean substitution was used to deal with the missing data, and a standardization procedure was used to rescale all attributes. Four different types of support vector machines (linear and polynomial) and one type of random forest (RF) were used to build the models. According to their research findings, the RF model had the best accuracy rate. A classification methodology for early diabetes detection using machine learning techniques is offered in a 2020 study [24,25]. This study aimed to produce results consistent with clinical outcomes using prominent traits. The information for this study came from a survey given to patients at the Diabetes Hospital in Sylhet, Bangladesh. A total of 520 examples with 16 variables are included in this dataset, all of which reflect symptoms associated with diabetes. Both positive (risk of diabetes) and negative (no risk of diabetes) diagnoses were determined using the two class features of the authors. We trained a multilayer perceptron (MLP), a radial basis function network (RBF), and a random forest (RF) to see which of these three classifiers was most effective in accurately predicting diabetes. Compared to other models, it was found that the RBF model was the most effective [26].

Some current studies have addressed the issue of using machine learning techniques to build prediction models for diabetic complications, in addition to the studies that predicted or diagnosed diabetes. The model established in 2020 by the authors in Ref. [27] to forecast hyperlipidemia, coronary heart disease, kidney disease, and eye disease as potential outcomes for people with diabetes is one such example. This research made use of a dataset consisting of 455. The dataset went through some selection and cleaning procedures that cut down on the number of records included in the model’s construction. The model was built using the iterative decision tree (ID3) algorithm. An accuracy of 92.35% was obtained using a 10-fold cross-validation procedure to assess the effectiveness of the suggested model. Especially when training on unbalanced data, the high accuracy score attained in this study is insufficient to evaluate the model’s performance. The key reason is that during training, the model can disregard a minority class and still produce accurate predictions for the majority class. To better anticipate the onset of retinopathy, neuropathy, and nephropathy in T2DM patients, the authors in a study conducted in 2018 [28] built various classification models such as LR, NB, SVMs, and random forest. The authors made their projections based on three different time frames: three, five, and seven years after the initial diabetic hospitalization. The dataset used to train the suggested models was compiled over a decade by researchers at the Istituto Clinico Scientifico Maugeri (ICSM), Hospital of Pavia, Italy. There are a total of 943 records here. They include topics such as gender, age, body mass index (BMI), time since diagnosis, hypertension, glycated hemoglobin (HbA1c), and smoking status. When dealing with missing data, the random forest method was used, and when dealing with uneven class sizes, oversampling the smaller group helped. The data collected showed that LR had the greatest accuracy score (77.7%).

## 3. The Proposed Methodology

**Figure 1:**
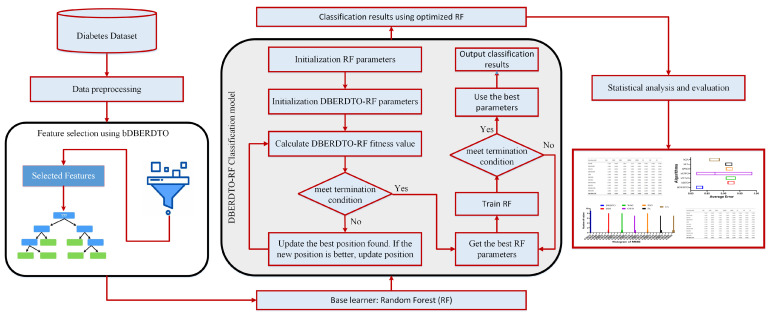
The architecture of the proposed system.

In this section, the proposed methodology is presented and explained. The methodology starts with data preprocessing, feature selection, and finally, diabetes classification. The key contribution of the proposed methodology is the proposed optimization algorithm. This optimization algorithm selects and optimizes the classifier parameters used in feature classification. The steps of the proposed methodology are shown in Figure 1, and these steps are detailed in the following sections.

### 3.1. Data Preprocessing

Data preprocessing is an essential step for cleaning, transforming, and preparing data to be used in modeling. One of the initial preprocessing steps is handling missing data. We can use mean, median, or mode imputation to fill in missing data values. Mathematically, we use the following equation to replace missing data with the mean value:

(1) $$\mathrm{Mean}=\frac{\sum_{i=1}^{n}x_{i}}{n}$$

Another preprocessing step is identifying and handling outliers. Mathematical functions such as the log and square root functions can be used to transform the data. This data preprocessing is critical to improving the accuracy of the models. The following equation demonstrates the log transformation of the data:

(2) LogTransform=log(x)

Normalization is also a crucial preprocessing step to ensure that all features are on the same scale. Min-max scaling is a common technique used to scale the data between 0 and 1, while the Z-score normalization scales the data using each feature’s mean and standard deviation. The following equation shows the min-max scaling of data:

(3) $$MinMaxScaling=\frac{x-x_{min}}{x_{max}-x_{min}}$$

### 3.2. Metaheuristic Optimization

Feature selection and the optimization of machine learning model parameters have benefited greatly from the rise in the popularity of metaheuristic optimization in recent years. This method shines when applied to situations with many moving parts or when extensive computational resources are needed to investigate all conceivable outcomes. Metaheuristic optimization algorithms excel in these situations because they can quickly and efficiently scour a large solution space for a desirable outcome. Non-differentiable, discontinuous, or multimodal objective functions are no problem for metaheuristic optimization. These functions frequently come up in feature selection and model parameter optimization tasks, where the goal is to discover the optimal set of features and model parameters to either reduce the error or maximize the accuracy of a machine learning model. In many fields, including medicine, business, and engineering, metaheuristic optimization techniques, including genetic algorithms, particle swarm optimization, and simulated annealing, have greatly affected feature selection and model parameter optimization [29,30]. The workings of nature or social systems, such as natural selection or swarm behavior, inspire these adaptive algorithms. Metaheuristic optimization is a robust method for feature selection and optimization of machine learning model parameters that can effectively deal with complex, non-differentiable, or multimodal objective functions. Discovering the optimal feature and parameter combinations that maximize accuracy for a given dataset can enhance performance and facilitate more informed decision-making across various domains [31,32,33,34,35].

### 3.3. Al-Biruni Earth Radius Optimization Algorithm

The Al-Biruni Earth Radius (BER) is an optimization technique that can enhance the search efficiency by dividing individuals in the search space into two groups that focus on exploration and exploitation [36]. This technique involves agents dynamically shifting the composition of subgroups to balance exploratory and exploitative pursuits. The exploration team, which constitutes 70% of the individuals, utilizes mathematical methods to look for promising new territory nearby. This is achieved through an iterative process of exploring alternatives until an optimal fitness level is achieved. Meanwhile, the exploitation team, consisting of 30% of individuals, focuses on exploiting the discovered optimal regions. The number of agents in both groups has increased to improve their global average fitness. To employ the BER optimization algorithm, each individual in the population is treated as a vector *S* representing the optimization parameter or features *d* in the search space of size $S_{d}\in R$. The fitness function *F* measures an individual’s success up to a given threshold. The optimization stages aim to probe populations and discover the S* value that maximizes fitness. The BER technique can be applied by specifying the fitness function, population size, dimension, and minimum and maximum acceptable solution sizes. Optimization algorithms aim to find the optimal solution within defined limits, and the BER technique can aid in achieving this goal. The BER technique has proven useful in optimizing machine learning models by improving search efficiency through a balance of exploration and exploitation. By dividing individuals into two groups and dynamically adjusting their composition, the BER technique can efficiently explore the search space and find the optimal combination of parameters and features. Furthermore, it can be easily employed by specifying the necessary parameters, making it a practical solution for optimizing complex problems.

The exploration process involves searching the search space for promising regions that can lead to finding the optimal solution. The lone explorer in the group looks for new locations to explore near their current location to move closer to the perfect solution. However, the effectiveness of exploration must be evaluated by exploring a variety of local possibilities and selecting the best ones. The BER technique utilizes the equations given below to achieve this goal:

(4) $$P\left(t+1\right)=P\left(t\right)+D\left(2r_{2}-1\right),D=r_{1}\left(P\left(t\right)-1\right)$$

The solution vector at iteration *t* is denoted by P(t), and the diameter of the circle within which the search agent searches for interesting regions is represented by *D*. The range of *x* in the coefficient vectors $r_{1}$ and $r_{2}$ is from 0 to 180, while the value of *h* is a scalar randomly chosen between [0,2]. The coefficients $r_{1}$ and $r_{2}$ can be obtained by solving the equation $r=h\frac{cos(x)}{1-cos(x)}$.

The team tasked with seizing opportunities must constantly improve existing methods. At the end of each cycle, the BER rewards those who have put the most effort toward achieving the highest fitness levels. The BER employs two distinct methods to achieve its exploitation goal, which we will discuss in detail. By using the equation provided below, we can take steps toward finding the best solution and move closer to the solution.

(5) $$P\left(t+1\right)=r^{2}\left(P\left(t\right)+D\right),D=r_{3}\left(L\left(t\right)-P\left(t\right)\right)$$

At each iteration *t*, P(t) is the solution vector, L(t) is the best solution vector, and *D* is the distance vector. $r_{3}$ is a random vector generated using the formula $r_{1}s=h\frac{cos(x)}{1-cos(x)}$. This formula governs the movement towards exploring the space around the best solution, which is the most promising of all possible solutions (leader). This encourages the exploration of solutions close to the ideal.

Furthermore, the BER uses another equation to optimize its search. The optimal solution is denoted by $P^{}\left(t\right)$, and the following equation guides the implementation of the optimal $P^{}$:

(6) $$P^{\prime }\left(t+1\right)=r\left(P^{*}\;\left(t\right)+k\right),k=1+\frac{2\times t^{2}}{Max_{iter}^{2}}$$

In this equation, *k* is a scalar factor that gradually increases with time, and $Max_{iter}$ is the maximum number of iterations. The best fitness value is compared between P(t+1) and $P^{\prime }\left(t+1\right)$ to choose the optimal $P^{*}$ implementation. If there is no improvement in fitness during the previous two iterations, the following equation is used to update the solution:

(7) $$P\left(t+1\right)=k*z^{2}-h\frac{cos(x)}{1-cos(x)}$$

In this equation, *z* is a random number in the [0,1] range. By constantly improving and updating its methods, the BER can effectively seize opportunities and optimize its search for the best solution.

### 3.4. The Dipper Throated Optimization Algorithm

The Dipper Throated Optimization (DTO) algorithm makes an innovative assumption that there are two groups of birds: the first group comprises swimming birds, and the second includes flying birds. These two groups cooperate in foraging food, mapped onto exploration and exploitation groups to find the best solution. The birds in these groups have positions and velocities that can be illustrated using matrices. The position matrix, P, contains the positions of the birds in each dimension, whereas the velocity matrix, *V*, includes the velocities of the birds in each dimension. Each bird’s fitness is measured by a fitness function, *f*, defined using the position matrix. During fitness evaluation, the mother bird has the highest fitness score, and the best solution is referred to as $P_{best}$. Common birds play the role of followers and are represented by $P_{nd}$, while the best solution in the search space is identified as $P^{*}$. The first approach of the DTO algorithm to track the swimming bird relies on the following equations to update the location and velocity of the birds in the population:

(8) $$X=P_{best}\left(i\right)-K_{1}.\left|K_{2}.P_{best}\left(i\right)-P\left(i\right)\right|$$

(9) Y=V(i+1)+P(i)

(10) $$\begin{array}{c} P\left(i+1\right)=\left\{\begin{array}{cc} X & \mathrm{if}\;R<0.5\\ Y & \mathrm{otherwise} \end{array}\right., \end{array}$$

(11) $$\begin{array}{cc} V\left(i+1\right)=K_{3}V\left(i\right) & +K_{4}r_{1}\left(P_{best}\left(i\right)-P\left(i\right)\right)+K_{5}r_{2}\left(P^{*}-P\left(i\right)\right) \end{array}$$

Here, *i* denotes the current iteration index, while i+1 represents the next iteration index. Equation X determines the change in the position of the bird from the best bird’s position. The Y equation updates the bird’s velocity based on its current velocity and position. In the P(i+1) equation, the bird’s position in the next iteration is updated based on the value of *R*. If *R* is less than 0.5, the bird’s position is updated using equation X. Otherwise, it is updated using equation *Y*. Finally, the V(i+1) equation updates the bird’s velocity by considering its current velocity, the distance between the best bird and the bird’s current position, and the distance between the $P^{*}$ and the bird’s current position. The constants $K_{1}$ to $K_{5}$ are coefficients that determine the impact of each factor on the bird’s position and velocity.

### 3.5. The Proposed Feature Selection Algorithm

Selecting the most pertinent features that contribute most to the classification accuracy is an important part of the diabetes classification process. Feature selection improves classification accuracy by decreasing the dimensionality of the, discarding non-essential or redundant features. This simplifies the model, enabling it to run more quickly and efficiently in real-time settings. Feature selection is the process of choosing which features are the most useful by evaluating them against a set of criteria, such as their correlation with the target variable or their ability to distinguish across classes. Feature selection can be accomplished in a number of ways, such as the filter, wrapper, or embedding approaches. To determine the importance of each feature outside of the context of the classification model, filter approaches use statistical tests or correlation coefficients. In contrast, wrapper techniques iteratively add and remove features based on the classification model’s assessment of their relative relevance. The feature weights of an embedded approach are learned directly from the data, and the method combines feature selection with the training process of the classification model. Feature selection is crucial in diabetes classification because it helps make classification models more accurate and interpretable. Multiple studies have demonstrated that by employing feature selection approaches, the dimensionality of the dataset can be drastically reduced without sacrificing accuracy in classification.

The accuracy and efficiency of classification models can be enhanced by feature selection, making it an essential strategy in diabetes classification. The scientists discovered that feature selection strategies increased classification performance compared to employing all the features, with mutual information-based feature selection yielding the best results. The scientists also stated that feature selection assisted in determining the most important traits associated with GDM, which could lead to improved diagnostic tools. Feature selection can assist in reducing the complexity of a dataset, making it more manageable for analysis and interpretation by highlighting the most important elements. In addition, feature selection can help zero in on the most informative features of the disease, which could lead to better, more interpretable diagnostic tools; for high-dimensional and complicated datasets as those used in diabetes classification, metaheuristic optimization algorithms have emerged as strong tools for feature selection. These algorithms take cues from natural occurrences or human behavior to get near-optimal results through efficient search space exploration by avoiding local optima. Feature selection for diabetes classification often uses metaheuristic algorithms, including genetic algorithms, particle swarm optimization, ant colony optimization, simulated annealing, and artificial bee colony. A fitness function, which might be based on classification accuracy, information gain, or some other criterion, can be used by these algorithms to search for an outstanding collection of features. Metaheuristic optimization can improve the speed and overcome the limits of other feature selection methods, such as wrapper, filter, and embedding approaches. When applied to feature selection for diabetes classification, metaheuristic optimization has been shown to be effective and can lead to the development of more accurate and resilient models.

When deciding whether or not a given feature is important, feature selection issues have a small search space consisting only of the binary values 0 and 1. To better accommodate the feature selection procedure, we present a binary version of the DBERDTO method, which transforms the continuous values produced by the original algorithm into binary [0, 1] values. The Sigmoid function is given by the following equation, which is used to execute the conversion to binary values.

(12) $$\begin{array}{cc} \; & S^{\left(t+1\right)}=\left\{\begin{array}{cc} 1 & \mathrm{if}\;Sigmoid(S_{best})\geq 0.5\\ 0 & otherwise \end{array}\right.,\\ \; & Sigmoid\left(S_{Best}\right)=\frac{1}{1+e^{-10(S_{Best}-0.5)}} \end{array}$$

where $S_{best}$ is the optimal solution for step *t* in the iterative process. Scaling the continuous values found in Algorithm 1 to the discrete range [0, 1] is the primary focus of the sigmoid function.
**Algorithm 1**: The proposed binary DBERDTO algorithm1:**Initialize** the configuration, parameters, and population of DBERDTO.2:**Binarize** the retrieved solutions using the sigmoid function3:**Evaluate** the objective function4:**Find** the best solutions.5:**Train** k-NN and calculate error6:**while** $t\leq Max_{iter}$ **do**7:   **Execute** the DBERDTO algorithm8:   **Binarize** the solutions using the sigmoid function9:   **Evaluate** the objective function10:   **Update** agents’ positions11:**end while**12:**Return** best binary solution

### 3.6. Objective Function

Using the proposed optimization approach, the following equation can be used to evaluate the received solution quality.

(13) $$F_{n}=\alpha Error\left(P\right)+\beta \frac{\left|S\right|}{\left|A\right|}$$

where *P* stands for some set of inputs to the model. The significance of the chosen features in the population is reflected by the values of α∈ [0, 1], β=1−α. The number of selected features, denoted by |S|, is smaller than the total number of features in the dataset, denoted by |A|. The optimal strategy is the one that uses the fewest features to make the most accurate classifications.

### 3.7. Optimizing the Hyperparameters of the Random Forest Classifier

The number of trees in the forest, the maximum depth of each tree, the minimum number of samples required to split a node, the minimum number of samples required to be at a leaf node, and the number of features to consider when looking for the best split, and the criterion used for splitting are all hyperparameters of the random forest classifier that can be optimized with metaheuristic optimization techniques. The number of trees is a critical hyperparameter since it controls how many individual decision trees are in the forest. A larger number of trees can improve the accurary of the model, but it will take longer to compute. The possible splits in each decision tree are constrained by its maximum depth, which might reduce the likelihood of overfitting. Each decision tree can be made as simple or complex as desired by adjusting the thresholds for when a node should be split and when a node should be considered a leaf. One of the most crucial hyperparameters in finding the optimal split is the number of features to evaluate. The random forest classifier uses a default setting that considers the square root of the total number of features. Both the Gini impurity and the entropy can be used as the criterion for splitting, and this hyperparameter can be tuned to boost the efficiency of the model. Finding the best settings for these hyperparameters helps boost the random forest classifier’s overall efficiency and precision, and metaheuristic optimization methods can help one achieve just that. These methods can be useful for locating optimal solutions that may not be obtainable using more conventional approaches since they search across a wide range of possible values for the relevant hyperparameters. These parameters are classified using the proposed optimization algorithm presented in Algorithm 2.
**Algorithm 2**: The proposed DBERDTO optimization algorithm1:**Initialize** DBERDTO population $P_{i}\left(i=1,2,\cdots ,d\right)$ with size *d*, iterations $Max_{iter}$, fitness function $F_{n}$, t=1, $n_{1}$, $n_{2}$, *a*, $r_{1}$, $r_{2}$, $r_{3}$, $r_{4}$, $r_{5}$2:**Calculate** fitness function $F_{n}$ for each $P_{i}$3:**Find** best solution as $P^{*}$4:**while** $t\leq Max_{iter}$ **do**5:   **if** t%2 == 0 **then**6:     **for** ($i=1:i\leq n_{1}$) **do**7:        **Update** $r_{1}=h\frac{cos(x)}{1-cos(x)}$8:        **Calculate** $D=r_{1}\left(P\left(t\right)-1\right)$9:        **Update** $P\left(t+1\right)=P\left(t\right)+D\left(2r_{2}-1\right)$10:     **end for**11:     **for** ($i=1:i\leq n_{2}$) **do**12:        **Calculate** $D=r_{3}\left(L\left(t\right)-P\left(t\right)\right)$13:        **Update** positions of best solution as $P\left(t+1\right)=r_{2}\left(P\left(t\right)+D\right)$14:        **Calculate** $k=1+\frac{2\;\times \;t^{2}}{Max_{iter}^{2}}$15:        **Investigate** area around best solution as $P^{\prime }\left(t+1\right)=r_{1}\left(P^{*}\left(t\right)+k\right)$16:        **Compare** P(t+1) and $P^{\prime }\left(t+1\right)$ to select best solution $P^{*}$17:        **if** best fitness is not changed for last two iterations **then**18:          **Mutate** solution as $P\left(t+1\right)=k*z^{2}-h\frac{cos(x)}{1-cos(x)}$19:        **end if**20:     **end for**21:   **else**22:     **for** ($i=1:i\leq n_{2}$) **do**23:        **if** ($r_{3}<0.5$) **then**24:          **Update** the individuals’ position as: $P\left(t+1\right)=P_{best}\left(t\right)-K_{1}.\left|K_{2}.P_{best}\left(t\right)-P\left(t\right)\right|$25:        **else**26:          **Update** the individuals’ velocity as: $V\left(t+1\right)=K_{3}V\left(t\right)+K_{4}r_{4}\left(P_{best}\left(t\right)-P\left(t\right)\right)+K_{5}r_{5}\left(P^{*}-P\left(t\right)\right)$27:          **Update** the individuals’ position as: P(t+1)=P(t)+V(t+1)28:        **end if**29:     **end for**30:   **end if**31:   **Update** the fitness function $F_{n}$ for each P(t)32:   **Update** BER and DTO parameters at t=t+133:**end while**34:**Return**$P^{*}$

### 3.8. The Classification Process Using Random Forest Classifier

The random forest classifier, following Algorithm 3, applies the bagging approach to each tree in the ensemble. Trees are fitted to this new, random sample instead of the training sample. One variable that can be learned automatically with the help of out-of-bag errors is the total number of trees used in the ensemble.
**Algorithm 3**: The classification of diabetes using random forest classification1:**Initialize** the selected feature vectors with n-samples and d-dimension2:**while** b≤N(baggingnumbers) **do**3:   **Get** a representative bootstrap ($X_{b},Y_{b}$)4:   **Develop** a random forest tree $T_{b}$ using ($X_{b},Y_{b}$)5:   **while** tree node size $\leq n_{min}$ **do**6:     **Get** random variables of size m7:     **Select** the most promising variables or split the m variables8:     **Break** the main node in the tree into two smaller nodes9:   **end while**10:   **Result** the trees ensemble as ${\left\{T_{b}\right\}}_{1}^{N}$11:**end while**12:**Return** the posterior $P\left(x\right)=Voting{P_{k}\left(x\right)}_{1}^{N}$, where the classification of the kth RF is denoted by $P_{k}\left(x\right)$

## 4. Experimental Results

In this section, we use the numerical figures, confusion matrix, and charts that resulted from using the proposed architecture to classify breast cancer. The experiments were run using the improved CBIS-DDSM public dataset. The cross-validation value is set at 5, and the training/testing split is 70:30. Several optimization techniques, such as the whale optimization algorithm (WOA) [37], genetic algorithm (GA) [38], particle swarm optimization (PSO) [39], grey wolf optimization (GWO) [40], Al-Biruni earth radius (BER) [36] optimization, and the proposed advanced BER algorithm, have been used to optimize the parameters of the CNN. Several trials, including a deep feature classification on the original dataset and a deep feature classification using the improved CNN, are used to arrive at the final results.

### 4.1. The Diabetes Dataset

**Figure 2:**
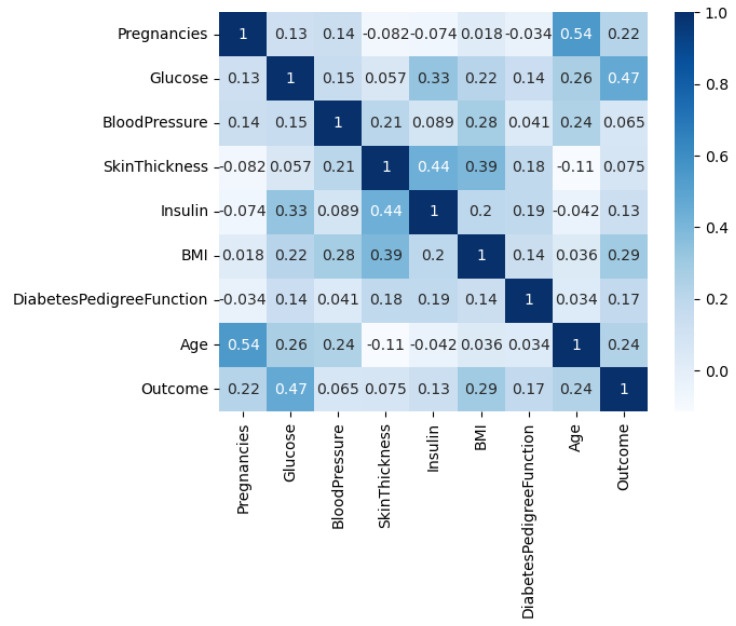
The correlation among the features of the diabetes dataset.

**Figure 3:**
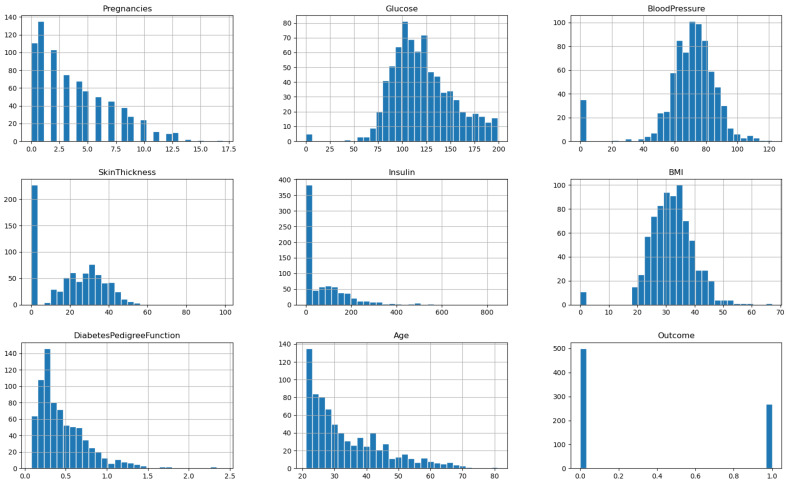
The histograms of the features of the diabetes dataset.

The Diabetes Dataset, denoted by D1 and available on Kaggle [41], contains data collected from 768 female patients of Pima Indian heritage residing in Arizona, USA. The dataset includes eight features or variables, including age, number of pregnancies, glucose level, insulin level, blood pressure, body mass index (BMI), diabetes pedigree function, and an outcome variable indicating whether or not the patient has diabetes. Age is a continuous variable representing the age of the patient in years. The number of pregnancies is an integer variable indicating the number of times the patient has been pregnant. Glucose level is a continuous variable representing the 2-h plasma glucose concentration in the patient’s blood. Insulin level is a continuous variable representing the serum insulin level in the patient’s blood. Blood pressure is a continuous variable representing the patient’s diastolic blood pressure in mm Hg. BMI is a continuous variable representing the body mass index of the patient. Diabetes pedigree function is a continuous variable representing the diabetes pedigree function for the patient, which provides information about the patient’s genetic predisposition for diabetes. Finally, the outcome variable is a binary variable indicating whether or not the patient has diabetes, with 1 representing the presence of diabetes and 0 representing the absence of diabetes. The Diabetes Dataset is a valuable resource for researchers and healthcare professionals interested in studying the risk factors associated with diabetes and developing better ways to prevent, manage, and treat the disease. By analyzing the relationships between these features and the outcome variable, researchers can identify important risk factors for diabetes and develop effective interventions and treatments to improve patient outcomes. The correlation among the features of the dataset is depicted in Figure 2, and the histogram of each feature vector is shown in the plots of Figure 3.

### 4.2. Feature Selection Evaluation Criteria

Table 2 provides the metrics by which the results have been evaluated. The performance of the suggested feature selection approach is measured against the metrics detailed in this table. The predicted values are denoted by $\hat{V_{n}}$, while the observed values are shown by $V_{n}$. The best solution at iteration *j* is represented by $S_{j}^{*}$, and the size of the best solution vector is denoted by $size(S_{j}^{*})$. *M* indicates the number of iterations of the proposed and other competing optimizers. A total of *N* points were used for the evaluation.

### 4.3. Classification Evaluation Criteria

The effectiveness of the proposed methods is measured using the benchmark metrics presented in Table 3. These metrics evaluate how well the proposed optimized CNN performs as a classification method. In the table, *M* represents the total number of iterations through an optimizer, $g_{j}^{*}$ represents the optimal solution for iteration *j*, and $size(g_{j}^{*})$ indicates the total length of the optimal solution vector. The number of data points in the test set, is denoted by *N*; the corresponding label, denoted by $C_{i}$, is determined by the classifier used. The number of features, denoted by *D*, and the class label, $L_{i}$, are two distinct quantities. True positive, true negative, false positive, and false negative abbreviations are TP, TN, FP, and FN, respectively.

### 4.4. Configuration Parameters

The configuration parameters of the employed optimization algorithms and the adopted machine learning models are presented in Table 4 and Table 5, respectively. In addition, the conducted experiments are operated based on 30 runs for the optimization algorithms, with 500 iterations in each run.

### 4.5. Results of Feature Selection

Using seven alternative metaheuristic optimization algorithms (bDBERDTO, bBER, bDTO, bPSO, bWAO, bGWO, and bFA), the authors report their findings about feature selection from diabetic features. The findings for each algorithm are summarized by their average select size. The average error results show the average error rate of each method, and they show that bDBERDTO has the lowest average error rate of 0.460, implying that the features selected by bDBERDTO can classify diabetes occurrences with a high degree of accuracy.

According to the results presented in Table 6, bWAO has the largest average select size (0.776), indicating that it has selected the most features on average. This suggests that bWAO is less efficient at reducing the dimensionality of the dataset, which may result in overfitting. The average fitness results show how each algorithm generally performs. Based on the results, bDBERDTO can be considered a powerful feature selection algorithm for diabetes classification, with an average fitness value of 0.523, which is significantly higher than the other algorithms. The highest fitness values show the greatest results for each algorithm. Based on the findings, bDBERDTO’s feature selection yields superior classification results to the other algorithms, with a best fitness value of 0.425. The lowest fitness values show the worst possible outcomes for each algorithm. Finally, the standard deviation fitness results show the variation in fitness values obtained by each algorithm, revealing that bFA has the worst fitness value of 0.606, indicating that bFA could not select a good subset of features that can perform well in diabetes classification. This suggests that the features chosen by bDBERDTO are more stable and can lead to consistent performance in diabetes classification, as evidenced by the lowest standard deviation of fitness values produced by bDBERDTO (0.346). As a whole, these findings are very suggestive of bDBERDTO’s potential as a feature selection method for diabetes classification.

The analysis of variance (ANOVA) is a statistical technique used to compare the means of two or more groups to establish statistical significance. The analysis was performed on a dataset of 69 samples from 7 groups, as presented in Table 7. With an F-value of 136.1 and a *p*-value of less than 0.0001, the results demonstrate that the Treatment factor significantly affected the data. This indicates a statistically significant change in the response variable due to the treatment and a difference in means between the groups. The error variance is estimated using the residual findings, which show the variation within each group. For this situation, 63 degrees of freedom (DF) were available, and the residual mean square was 0.00179. This suggests a tiny amount of diversity inside each group, but the variation across groups is much more significant. The results in the total column reflect the sum of all possible differences in the data, both between and within groups. The total number of degrees of freedom was 69, and the sum of squares (SS) was 0.02502. The analysis of variance test results indicates that the differences between the treatment groups are statistically significant and not coincidental. The relationship between therapies and the response variable can be better understood, and judgments regarding which treatment is most effective can be made with the help of this data.

A non-parametric alternative is the Wilcoxon signed-rank test to compare two related samples. For each of the seven feature selection techniques (bDBERDTO, bBER, bDTO, bPSO, bWAO, bGWO, and bFA), the test is used to compare the theoretical median values (assumed to be zero) with the actual median values presented in Table 8. Ten examples of each technique are used in the test. Each observation is given a rank, with higher ranks going to observations above the median and lower ranks going to observations below the median. These ranks are the “sum of signed ranks” (W). In this situation, the median values are continuously greater than the theoretical median of zero, as the sum of signed rankings is 55 across all 7 approaches. The total rankings for all “positive” and “negative” observations are summed to get the “sum of positive ranks” and “sum of negative ranks”, respectively. In this situation, all methods have median values greater than the theoretical median of zero, as seen by the total of the positive ranks being 55. No actual median values are below the theoretical median, as the sum of negative ranks is 0 for all techniques. Suppose that the null hypothesis (which here states that the observed median values are not significantly different from the theoretical median of zero) is correct. In that case, the “*p*-value” (two-tailed) is the probability of obtaining a test statistic as extreme as or more extreme than the observed test statistic. For all seven approaches, the P value is less than 0.002, suggesting sufficient evidence to reject the null hypothesis and infer that the observed median values differ substantially from the theoretical median. If the *p*-value was calculated exactly or estimated, as the information in the “Exact or estimate?” field. Here, the calculated P values are accurate. If you want to know if your results are statistically significant at the 0.05 level, enter that number into the “Significant (alpha = 0.05)?” column. Given that the P value is less than 0.05, these findings are statistically significant at the 5% level of confidence. Finally, the discrepancy between the observed median values and the ideal zero median is displayed in the “Discrepancy” section. In this category, the numbers represent the reported medians for each technique. The results show that, similar to every other feature selection approach evaluated, the suggested method (bDBERDTO) deviates significantly from the expected zero medians.

**Figure 4:**
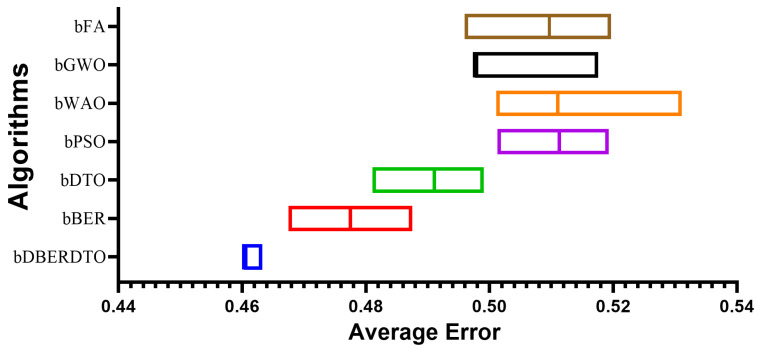
The average error of the proposed feature selection method results.

In Figure 4, the proposed method, bDBERDTO, has the lowest average error in a plot comparing the average errors achieved by other feature selection techniques. The plot also included the other methods bFA, bGWO, bWOA, bPSO, bDTO, and bBER, but none of them could match the performance of bDBERDTO. The plot shows that the proposed method is quite good at picking out the most important features in a dataset, which improves the overall performance of the system. It’s important to remember that even the other methods managed to attain low average mistakes, showing that they are not without merit. However, the recommended strategy emerges as the undisputed victor in this comparison. The ramifications of these findings for professionals in domains that use feature selection techniques to boost model performance are substantial. Compared to popular feature selection approaches, bDBERDTO is expected to produce even better outcomes.

**Figure 5:**
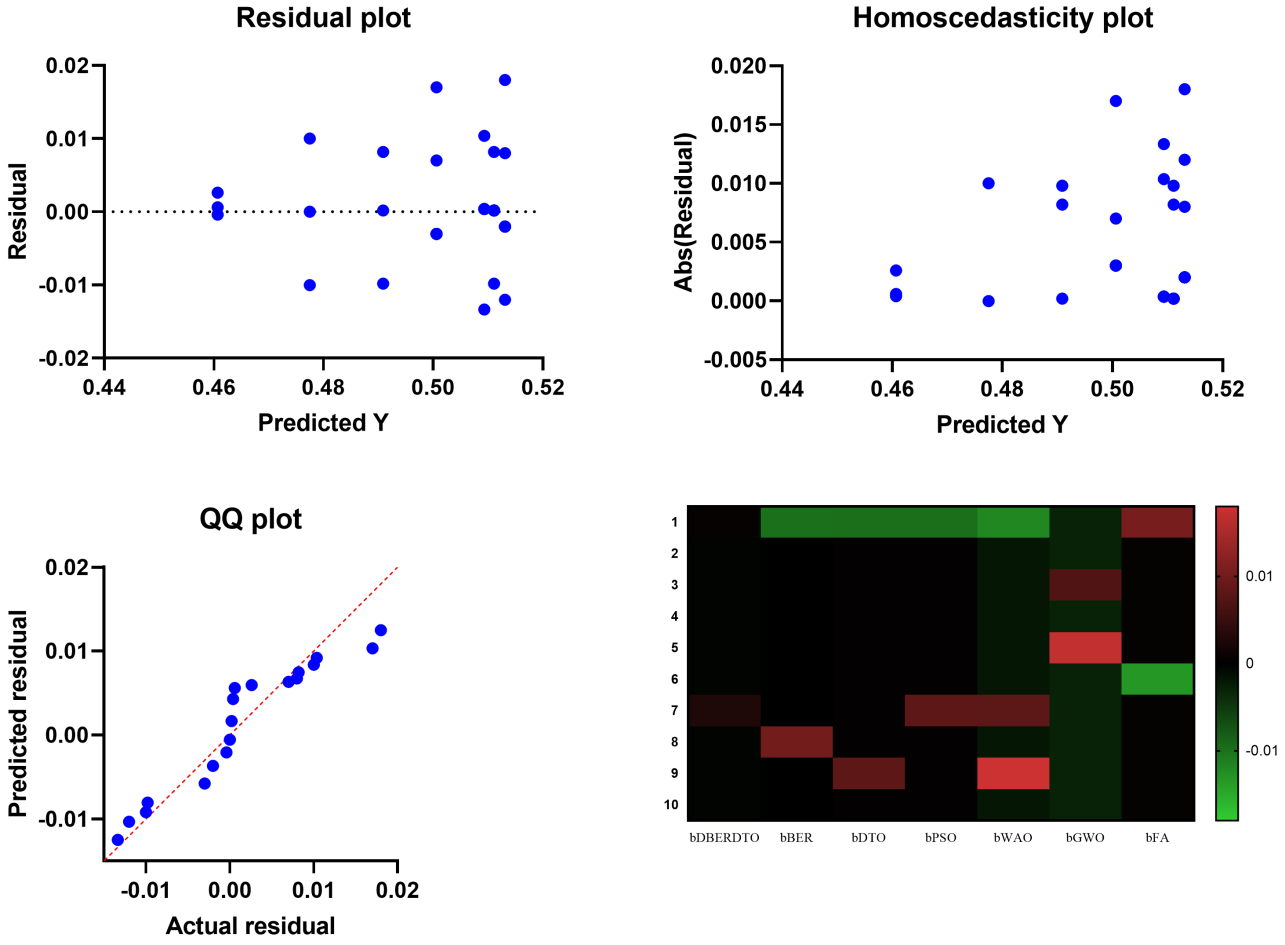
Visualizing the ANOVA test applied to the proposed feature selection method results.

In Figure 5, the residual, homoscedasticity, quartile-quartile (QQ), and heatmap plots are used to examine the ANOVA outcomes of the suggested feature selection technique. Residual and homoscedasticity plots are used to verify that the errors have the same variance and that the data is normally distributed. QQ plots are used to evaluate the normality of the residuals, while heatmap plots are used to see the connections between the chosen features. Providing that the assumptions are met, the residual plot, which displays the discrepancy between the observed and anticipated values, should exhibit no discernible pattern. The homoscedasticity plot’s residuals should be randomly dispersed around the horizontal line to ensure that the error variance is the same across all predictor variables levels. A straight line in the QQ plot indicates properly distributed residuals. We can gain insight into potential multicollinearity issues by displaying highly correlated features in a heatmap. These plots, when combined, give a thorough picture of the ANOVA results of the proposed feature selection approach, allowing practitioners to evaluate the assumptions and see any problems that may need fixing before interpreting the results.

Different classification algorithms were evaluated on a diabetes dataset consisting of patient medical records and other parameters used to determine diabetes risk, and the findings are presented in Table 9. Some performance indicators are *p*-values, F-scores, precision, sensitivity, and specificity. The *p*-value measures how likely it is to find a test statistic that is at least as outlandish as the one found in the dataset. The F-score examines how well true positive and false positive rates are balanced. F is the harmonic mean of precision and recall. The accuracy of a prediction system is measured by how many of those forecasts come true. An accurate positive rate is known as sensitivity, and an accurate negative rate is known as specificity. With an accuracy of 0.813, a sensitivity of 0.859, a specificity of 0.741, an F-score of 0.768, and a *p*-value of 0.840, random forest (RF) outperformed the other classification algorithms. Logistic regression (LR) came in second, scoring an F-score of 0.760 and a *p*-value of 0.800 with an accuracy of 0.787, sensitivity of 0.870, and specificity of 0.655. The support vector machine (SVM) results were similarly quite good: an F-score of 0.838, a *p*-value of 0.761, a sensitivity of 0.935, and a specificity of 0.534. Regarding accuracy and F-scores, k-nearest neighbors (KNN) and decision tree (DT) were less effective than RF, LR, and SVM. Stochastic Gradient Descent (SGD) performed the worst in accuracy, sensitivity, and F-score compared to other classifiers. These findings indicate that RF, LR, and SVM are superior to KNN and DT when predicting diabetes from the provided information.

After applying feature selection, the classification outcomes for a diabetes dataset are presented in Table 10. *p*-values, F-scores, degrees of precision, and specificity are all reported. Stochastic Gradient Descent (SGD), Decision Trees (DT), K-Nearest Neighbors (KNN), Gaussian Naive Bayes (GNB), Support Vector Machines (SVM), Logistic Regression (LR), and Random Forest (RF) were all employed as classification methods. A small *p*-value (0.588) indicates that the features used to train the SGD classifier are not very predictive of the outcome. Moderate F-score and accuracy (0.657) are accompanied by high sensitivity (0.909) and specificity (0.432) but low F-score (0.714) and accuracy (0.657). The *p*-value for the DT classifier is somewhat higher (0.682) than the *p*-value for SGD, but neither is statistically significant. Compared to SGD, the F-score and accuracy are better (0.779 and 0.73), although the sensitivity and specificity are the same (100). The KNN classifier’s greatest *p*-value (0.769) indicates that the selected features are more important in predicting the outcome than any other classifiers. High levels of sensitivity (0.943) and specificity (0.545) are accompanied by a high F-score (0.847) and high levels of accuracy (0.791). The GNB classifier has a moderately low *p*-value (0.806) and a high F-score (0.87) and accuracy (0.819). In contrast to the moderate specificity (0.6), the sensitivity is relatively high (0.943). With a *p*-value of 0.857, SVM is the second most accurate classifier, suggesting that the features used to make the prediction are crucial. Very high sensitivity (0.909) and moderate specificity (0.762) are accompanied by a high F-score (0.882) and high accuracy (0.852). High *p*-value (0.851), high F-score (0.889), and high accuracy (0.853) are all features of the LR classifier. Although the specificity is at 0.72, the sensitivity is very high at 0.93. Last but not least, the RF classifier excels in all three metrics studied here: F-score (0.909), accuracy (0.885), and sensitivity (0.909). The sensitivity (0.842) and the specificity (0.909) are pretty good. In conclusion, the KNN and RF classifiers achieved the highest F-scores, accuracy, sensitivity, and specificity following feature selection. It is important to highlight that the classifier selection is problem- and data-specific and that additional investigation may be required to identify the optimal model.

The analysis presented in Table 11 provides multiple statistical indicators of diabetes categorization model performance. There is valuable insight to be gleaned from each metric concerning the precision and consistency of the model’s predictions. According to the first metric, “Number of values”, there were ten occurrences across all seven categories. Although their precise meanings aren’t specified, we can infer that they correspond to different aspects of the model’s performance or the data used to train and evaluate it. Both the minimum and maximum values for a given category are indicated by the respective “Minimum” and “Maximum” indicators. For instance, the first group ranges from 0.986 to 0.992 for its values. Outliers can be found, and the range of values for each category is determined with these metrics. In order to determine how far any number can go, we can use the “Range” metric. There was a wide disparity between the highest and lowest values, as seen by the range of 0.030 in the fifth group. Data on the distribution of values within each category is made available through the “Percentile” measurements. For example, the “Median” value divides the data in half, while the “25% Percentile” reflects the number below which 25% of the data falls. The “75% Percentile” indicates the figure below which 75% of the data falls. These metrics can help show where the data is most and least concentrated and whether or not it is biased. Similar information is provided by the “10% Percentile” and “90% Percentile” measures, but for the 10th and 90th percentiles of the data, instead of the median and interquartile ranges. Information about the results’ reliability is provided by using the “Actual confidence level”, “Lower confidence limit”, and “Upper confidence limit” metrics.

These estimates are based on a confidence interval, a range of numbers thought to contain the actual value of the population parameter under study. With a confidence level of 97.85%, the actual number is likely inside the estimated margin of error. Indicators of central tendency, dispersion, and variability in data are provided by the “Mean”, “Std. Deviation”, “Std. Error of Mean”, and “Coefficient of Variation” measurements. The mean is the value that is most often encountered, whereas the standard deviation and standard error of the mean tell us about the variation in the data and the precision with which we may estimate the mean. The coefficient of variation is a measure of variability independent of measurement units and can be used to assess similarities and differences in data distribution across distinct groups. If the data is highly skewed, “Geometric Mean” measure can be used as an alternate central tendency measure. The “Geometric SD Factor” measures the dispersion of the data. At the same time, the “Lower 95% CI of geo. means” and “Upper 95% CI of geo. mean” give the minimum and maximum values of the 95% confidence interval for the geometric mean, respectively. The “Harmonic Mean” measures offer a different kind of central tendency metric that might be helpful when working with severely skewed data. Lower and upper limits of the 95% confidence interval for the harmonic mean are provided under the headings “Lower 95% CI of harm. mean” and “Upper 95% CI of harm. mean”, respectively. In cases where the data is highly skewed, the “Quadratic Mean” measurements provide yet another alternative central tendency measure. The lower and upper bounds of the 95% confidence interval for the quadratic mean are provided by the “Lower 95% CI of the quad. mean” and “Upper 95% CI of the quad. mean” headings, respectively.

One way to compare and contrast many groups or treatments is via an analysis of variance (ANOVA) test, which is presented in Table 12. The primary sections of the ANOVA table are labeled “Treatment”, “Residual”, and “Total”, respectively. The term “treatment” describes the differences in means between the various groups. The term “residual” is used to describe the ambiguous variation or inaccuracy that exists among the groups. What we mean by “total” here is the sum of the squares of all the observations or the entire amount of variability. The following is a breakdown of the metrics in each component of the ANOVA table:

Treatment = ( 0.02327 and 6 and 0.003878 and F (6, 63) = 211.8 and *p* < 0.0001):

- SS (sum of squares) = 0.02327, representing the treatment groups’ variability;
- DF (degrees of freedom) = 6, representing the number of groups being compared minus one;
- MS (mean square) = 0.003878, representing the treatment group variance;
- F (DFn, DFd) = 211.8, representing the F-statistic or the variance ratio between the treatment groups to the variance within the treatment groups;
- *p*-value < 0.0001 represents the probability of observing such an extreme F-statistic or more powerful under the null hypothesis that no difference exists between the treatment groups.

Residual = ( 0.00115 and 63 and 0.000018):

- SS = 0.00115 represents the unexplained variability or error within the groups;
- DF = 63, representing the total number of observations minus the number of groups being compared;
- MS = 0.000018, representing the treatment group variance.

Total = ( 0.02442 and 69):

- SS = 0.02442 represents the total variability or sum of squares of all the observations;
- DF = 69, representing the total number of observations minus one.

With an F-statistic of 211.8 and a *p*-value of 0.0001, the variance analysis shows a statistically significant difference between the treatment groups. The ANOVA table’s Treatment column indicates more variation between treatment groups than within them. In contrast, the Residual column suggests some variation or error within the groups that cannot be accounted for by the other two columns.

The Wilcoxon signed-rank test is a useful non-parametric option when comparing two groups with some features. The evaluation in this scenario aims to assess the relative merits of several machine learning approaches as presented in Table 13. If there is no difference between the two approaches, then the theoretical median is a vector of zeros. The empirical median equals the mean of the performance gaps between the various approaches and the theoretical median. Values equal the total number of test observations. In this scenario, there are 10 data points for each approach under consideration. Differences between the true and theoretical median can be quantified by adding up the signed rankings (W). When the rank is higher than zero, the real median exceeds the theoretical median; when the rank is lower than zero, the actual median falls short of the theoretical median. Considering that 55 is the sum of positive and negative ranks, we may conclude that all deviations from the theoretical median are positive. A test’s statistical significance is shown by its *p*-value. Evidence exists to reject the null hypothesis if the *p*-value is smaller than the significance level (alpha). All of the *p*-values shown are smaller than 0.05, indicating that there is indeed a statistically significant difference between the two approaches. The *p*-values are determined precisely, rather than being approximated, thanks to the accurate nature of the test. Positive signed ranks and a smaller difference between the actual and theoretical medians show that the proposed approach (DBERDTO + RF) outperforms the other methods by a wide margin.

**Figure 6:**
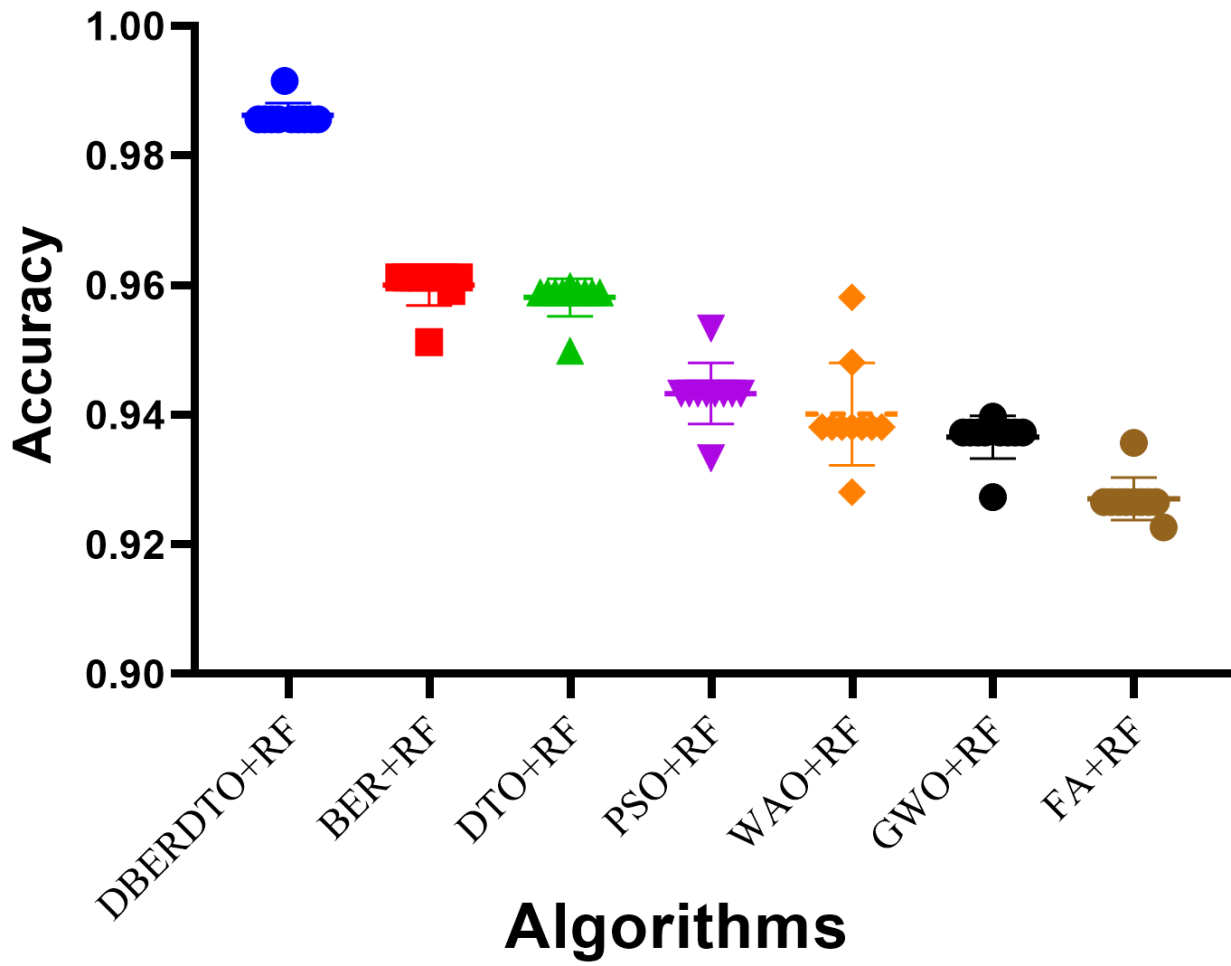
The accuracy achieved by the optimized RF classifier compared to other optimization methods.

The plot shown in Figure 6 comparing the classification accuracy attained by several strategies for diabetes case classification reveals that the proposed DBERDTO + RF strategy delivers the maximum accuracy. In addition to BER + RF, DTO + RF, PSO + RF, WOA + RF, GWO + RF, and FA + RF, we plotted these and other categorization approaches to see how they stacked up. The scatter plot shows that the proposed technique is very good at correctly categorizing instances of diabetes. Classification accuracy was still rather good for the remaining approaches, indicating that they had merit in their own right. Although the offered solution is not the only viable option, it is the most advantageous in this scenario. As correct diabetes case classification is crucial for both diagnosis and treatment, these findings have substantial implications for medical professionals. To get even better results than with other standard classification approaches, users can try the DBERDTO + RF strategy. The high accuracy of the proposed method has the potential to improve the diagnosis and treatment of diabetes, which in turn will enhance the lives of those who suffer from the disease.

**Figure 7:**
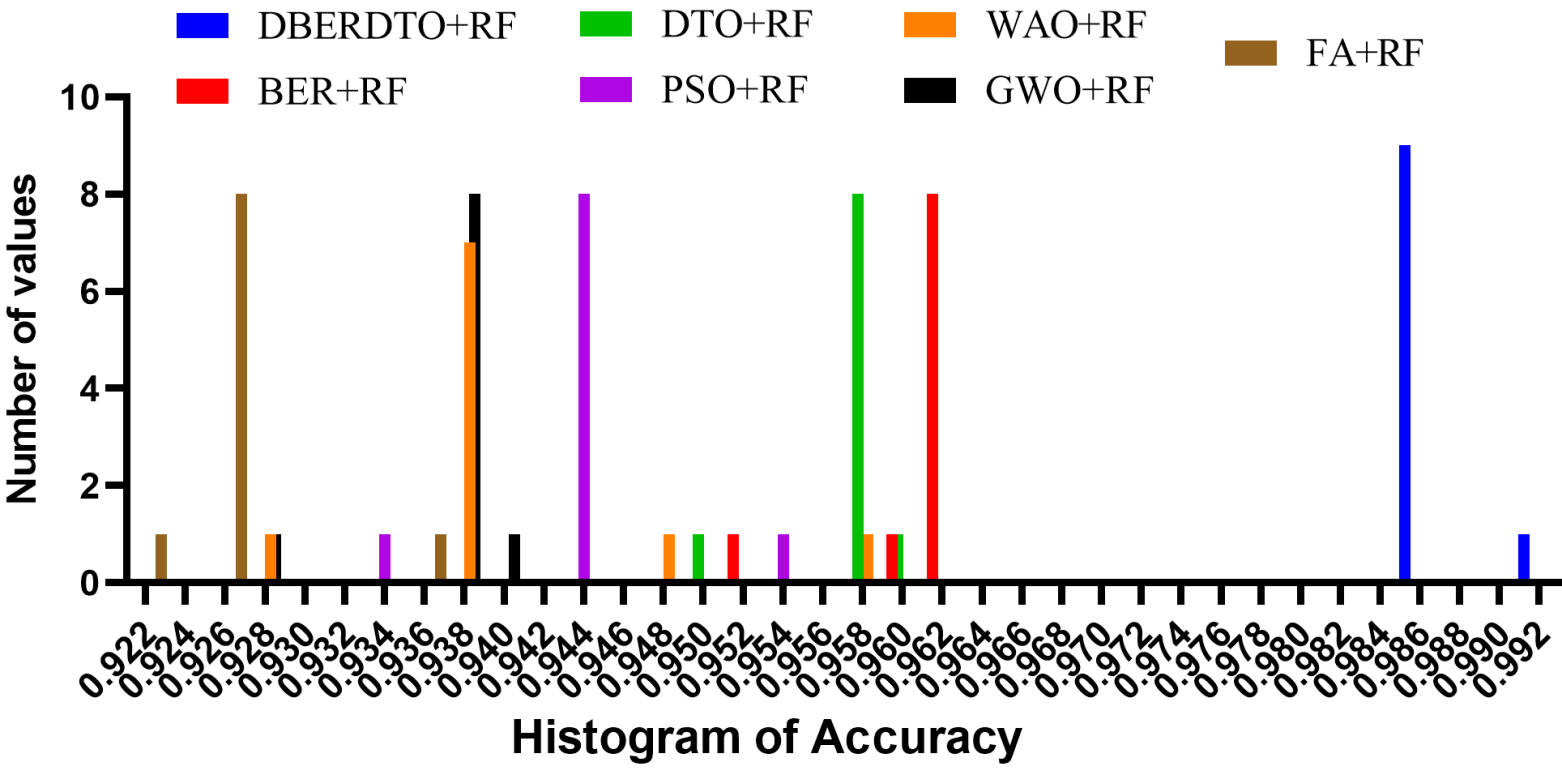
The accuracy histogram achieved by the optimized RF classifier compared to other optimization methods.

**Figure 8:**
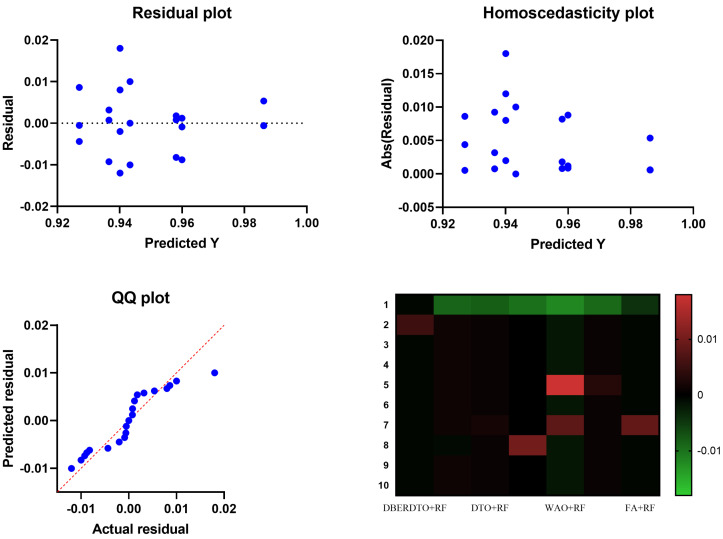
Visualizing ANOVA test results when applied to the results of the optimized RF classifier.

As shown in Figure 7, the proposed DBERDTO + RF strategy achieves the highest accuracy compared to other methods in a histogram plot, comparing the classification accuracy attained by various approaches in classifying diabetes cases. Each method’s classification accuracy is displayed as a histogram, with the height of each bar indicating the frequency with which that accuracy number was obtained. The histogram shows that the most frequent accuracy values are concentrated at the maximum accuracy attained by the DBERDTO + RF method, confirming the superiority of the suggested method. This visualization helps practitioners select the most efficient way for their specific use case and further demonstrates the proposed strategy’s superiority in accurately identifying diabetes patients. On the other hand, the plots shown in Figure 8 show the significance of the proposed approach in classifying diabetes cases compared to the other methods.

### 4.6. Discussion

The Dynamic Al-Biruni Earth Radius and Dipper Throated Optimization (DBERDTO) algorithm is used in the proposed method for diabetes classification, and it shows promise in both feature selection and classification. The method outperforms competing modern optimization techniques by using the binary version of DBERDTO for feature selection and the continuous version for optimizing the parameters of the random forest classifier. Feature selection plays a crucial role in selecting the most informative features for a classification task. When applied to feature selection, the binary version of DBERDTO shows superior results, demonstrating its efficacy in discovering important features for diabetes categorization. DBERDTO effectively searches the feature space and picks a subset of features that maximizes the classification performance by utilizing the algorithm’s distinctive exploration and exploitation methodologies. DBERDTO’s flexibility in balancing worldwide exploration with local exploitation is one of its greatest strengths. The algorithm can successfully navigate the feature space since it considers the variety of features and their local correlations. Therefore, the proposed method benefits from a strong feature subset that captures the crucial discriminatory data about diabetes. To maximize the performance of a random forest classifier, one should fine-tune its settings. This method successfully tunes the parameters of a random forest using a continuous version of DBERDTO. DBERDTO optimizes the random forest parameters to improve the classification performance by adjusting its search behavior dynamically throughout the optimization process. Random forests are an ensemble classifier combining the predictive power of several decision trees. Selecting the correct hyperparameters, such as the number of trees, maximum depth, and split criterion, is crucial to the performance of a random forest. The proposed method employs DBERDTO, automatically tweaking these settings to produce optimal classification outcomes.

Compared to other modern optimization techniques, the proposed method based on DBERDTO performs better in feature selection and classification tasks for diabetes classification. There are several important reasons why this strategy produces better results. The first distinguishing feature of DBERDTO is its capacity to efficiently search the solution space and settle on good solutions by modifying its search behavior. This flexibility allows the algorithm to locate optimal feature subsets and parameter combinations despite potential optimization problems. The proposed method provides a complete optimization framework because it combines the binary and continuous forms of DBERDTO. The strategy takes advantage of the strengths of both the binary and continuous versions by using the former for feature selection and the latter for optimizing parameters. The method enhances computational efficiency and generalization performance by picking the most informative features within the dataset. DBERDTO’s feature subset selection collects the most important discriminatory information, increasing classification accuracy. The efficiency of the proposed method is further demonstrated by the superiority of the classification results achieved using the method. DBERDTO-optimized random forest parameters improve the accuracy, sensitivity, and specificity of diabetes detection classification. Accurate diabetes classification is essential for better patient treatment; these enhanced performance indicators are a key part of that. The DBERDTO algorithm, used in the proposed method for diabetes classification, shows substantial advantages over other contemporary optimization techniques. DBERDTO’s binary implementation allows for enhanced feature selection, accurately determining the most important features. The classification accuracy, sensitivity, and specificity are all improved in the continuous version by optimizing the random forest settings. The proposed approach is superior because of the DBERDTO algorithm’s novel features, such as its dynamic search behavior and integration of global exploration and local exploitation tactics. These features help it identify the best possible classification methods for diabetic patients.

The selected features obtained through the proposed feature selection method demonstrate their potential to improve the performance of the optimized random forest classifier on the adopted dataset. The results indicate that these selected features possess discriminative power and contribute significantly to the classification task. By focusing on relevant features, the classifier can make more accurate classifications and achieve higher performance metrics such as precision, recall, and accuracy. The implications of diabetes diagnostics are significant in terms of public health and individual well-being. Accurate and reliable diagnostic methods can aid in the early detection of diabetes, enabling timely intervention and management. With the selected features improving the performance of the classifier, it suggests that these features have strong associations with diabetes-related patterns or risk factors. This insight can help healthcare professionals and researchers better understand the underlying factors contributing to diabetes and develop targeted interventions. Moreover, the improved performance of the classifier implies the potential for creating efficient and reliable diagnostic tools for diabetes. By identifying the most informative features, future diagnostic models and algorithms can be developed that focus on these key factors. This can lead to the development of cost-effective, non-invasive, and accessible diagnostic methods, facilitating the early detection and proactive management of diabetes. However, it is essential to note that the discussion of the selected features and their potential performance improvements should be interpreted within the context of the adopted dataset and the applied feature selection methods. To validate these findings on larger and more diverse datasets to ensure the generalizability and robustness of the selected features in different populations and settings, three other datasets and the achieved results and findings are discussed in the Appendix A.

## 5. Conclusions and Future Perspectives

In this paper, we proposed a novel metaheuristic optimization-based method for improving diabetes classification. A novel feature selection algorithm is developed using a dynamic Al-Biruni earth radius and throated dipper optimization (DBERDTO) algorithm. Following feature selection, the proposed DBERDTO is used to optimize the parameters of a random forest classifier before applying it to the dataset. To demonstrate the efficacy and superiority of the suggested methodology, it is compared and evaluated against state-of-the-art optimization techniques and machine learning models. The proposed method can classify diabetes cases with an overall accuracy of 98.6%. Analysis of variance (ANOVA) and Wilcoxon signed-rank tests were performed, among others, to gauge the significance and difference of the proposed approach. The outcomes of the tests corroborated the predicted results. The proposed approach and its possible application to other medical datasets will be studied in greater depth in future research. In addition, there are a variety of data-balancing methods that can deal with outliers and will be considered in future work.

## Figures and Tables

**Table 1:** Review of the recent methods used in diabetes classification.

Paper	Method	Features	Results	Limitations
[18]	KNN, naive Bayes (NB), C4.5 decision tree, SVM	Uses relevant kernel functions to resolve various issues. When dealing with unstructured data, SVM excels. In addition, it can produce useful outcomes even when only partial data is available.	The C4.5 decision tree model has the highest accuracy (73%) compared to the other classifiers studied.	Using large datasets in the training process is time-consuming. Since their relative importance is not fixed, each input variable contributes in a unique way to the final result.
[20]	Artificial neural network (ANN)	The ANN is intuitive and quickly adapts to new situations. It is helpful for addressing issues where noisy input data is present.	There was a 93% success rate for the ANN model.	Knowing the required neurons and layers is difficult, and learning can be slow.
[21]	Random forest, naive Bayes, XG Boost, decision trees, KNN and AdaBoost	The use of KNN is simple. It is optimal for datasets with several classes. Training is efficient, producing high-quality outcomes quickly.	AdaBoost and XG Boost were the most effective models in an ensemble setting. Compared to other research, this one had the highest area under the curve (AUC) score, reaching 0.95.	Memory limitation and a long time to find the nearest neighbors in large datasets.
[21]	Random forest, SVM, logistic regression (LR), and linear discriminant analysis (LDA)	Can be applied to two or multiple classes. The implementation is simple, and the classification is fast.	The RF model performed best, with an accuracy score of 82%.	Complex nonlinear data is beyond its capabilities. LDA distributions deviate significantly from Gaussianity. If the variance of the data is used as a discriminator instead of its mean, LDA will not work.

**Table 2:** Evaluation metrics used in assessing the proposed feature selection method.

Metric	Value
Mean	$\frac{1}{M}\sum_{i=1}^{M}S_{i}^{*}$
Best Fitness	$min_{i=1}^{M}S_{i}^{*}$
Average Error	$\frac{1}{M}\sum_{j=1}^{M}\frac{1}{N}\sum_{i=1}^{N}mse\left(\hat{V_{i}}-V_{i}\right)$
Worst Fitness	$max_{i=1}^{M}S_{i}^{*}$
Average fitness size	$\frac{1}{M}\sum_{i=1}^{M}size\left(S_{i}^{*}\right)$
Standard deviation	$\sqrt{\frac{1}{M-1}\sum_{i=1}^{M}{\left(S_{i}^{*}-Mean\right)}^{2}}$

**Table 3:** Evaluation metrics used in assessing the optimized random forest classifier.

Metric	Value
Accuracy	$\frac{TP+TN}{TP+TN+FP+FN}$
Sensitivity	$\frac{TP}{TP+FN}$
Specificity	$\frac{TN}{TN+FP}$
F1-Score	$\frac{TP}{TP+0.5(FP+FN)}$
N-value	$\frac{TN}{TN+FN}$
P-value	$\frac{TP}{TP+FP}$

**Table 4:** Configuration parameters of the employed optimization algorithms.

Algorithm	Parameter	Values	Algorithm	Parameter	Values
BER [36]	Iterations	80	GWO [40]	*a*	2 to 0
	Mutation probability	0.5		Iterations	80
	Exploration percentage	70		Wolves	10
	k (decreases from 2 to 0)	1	WOA [37]	*r*	[0, 1]
DTO [42]	Iterations	80		Iterations	80
	Number of runs	30		Whales	10
	Exploration percentage	70		*a*	2 to 0
GA [38]	Cross over	0.9	PSO [39]	Acceleration constants	[2, 2]
	Mutation ratio	0.1		Inertia $W_{max}$, $W_{min}$	[0.6, 0.9]
	Mechanism	Roulette wheel		Particles	10
	Agents	10		Iterations	80
	Iterations	80			

**Table 5:** Configuration parameters of the baseline classification models.

Model	Parameter	Value
SGD [43]	loss	hinge
	penalty	l2
DT [44]	splitter	‘best’
	min_samples_split	2
	criterion	‘gini’ to 0
	min_samples_leaf	1
KNN [45]	n_neighbors	5
	weights	‘uniform’
	leaf_size	30
	p	2
GNB [46]	Likelihood of features	Gaussian
SVM [47]	C	1
	kernel	‘rbf’
	penalty	‘l2’
	tol	1.0 × 10${}^{-4}$
LR [48]	solver	‘svd’
	tol	1.0 × 10${}^{-4}$
	shrinkage	[0–1]
RF [49]	max_depth	2
	random_state	0

**Table 6:** Evaluation of the results of the proposed feature selection method.

D1	bDBERDTO	bBER	bDTO	bPSO	bWAO	bGWO	bFA
Average error	0.460	0.477	0.491	0.511	0.511	0.498	0.510
Average Select size	0.413	0.613	0.555	0.613	0.776	0.536	0.648
Average Fitness	0.523	0.540	0.551	0.538	0.546	0.546	0.590
Best Fitness	0.425	0.460	0.454	0.518	0.510	0.524	0.509
Worst Fitness	0.524	0.527	0.569	0.586	0.586	0.600	0.606
Std Fitness	0.346	0.350	0.352	0.350	0.352	0.351	0.387

**Table 7:** Analysis of variance (ANOVA) of the feature selection results. In this table, SS denotes (sum of squares), DF (degrees of freedom), DFn denotes DF numerator and DFd denotes DF denominator. MS (mean square).

D1	SS	DF	MS	F (DFn, DFd)	*p*-Value
Treatment	0.02323	6	0.003871	F (6, 63) = 136.1	*p* < 0.0001
Residual	0.00179	63	0.00002845		
Total	0.02502	69			

**Table 8:** Wilcoxon signed rank test of the proposed feature selection method.

D1	bDBERDTO	bBER	bDTO	bPSO	bWAO	bGWO	bFA
Theoretical median	0	0	0	0	0	0	0
Actual median	0.4603	0.4775	0.4911	0.5113	0.5111	0.4976	0.5097
Number of values	10	10	10	10	10	10	10
Sum of signed ranks (W)	55	55	55	55	55	55	55
Sum of positive ranks	55	55	55	55	55	55	55
Sum of negative ranks	0	0	0	0	0	0	0
P value (two tailed)	0.002	0.002	0.002	0.002	0.002	0.002	0.002
Exact or estimate?	Exact	Exact	Exact	Exact	Exact	Exact	Exact
Significant (alpha = 0.05)?	Yes	Yes	Yes	Yes	Yes	Yes	Yes
Discrepancy	0.4603	0.4775	0.4911	0.5113	0.5111	0.4976	0.5097

**Table 9:** Evaluation of the classifiers results before applying feature selection.

D1	*p*-Value	*p*-Value	F-Score	Accuracy	Sensitivity	Specificity
SGD	0.729	0.500	0.441	0.533	0.380	0.776
DT	0.756	0.730	0.578	0.680	0.707	0.638
KNN	0.725	0.763	0.625	0.693	0.804	0.517
GNB	0.813	0.809	0.695	0.767	0.804	0.707
SVM	0.761	0.839	0.838	0.780	0.935	0.534
LR	0.800	0.833	0.760	0.787	0.870	0.655
RF	0.840	0.849	0.768	0.813	0.859	0.741

**Table 10:** Evaluation of the results of the classifiers after applying the proposed feature selection method and before optimizing the parameters of the classifiers.

D1	*p*-Value	*p*-Value	F-Score	Accuracy	Sensitivity	Specificity
SGD	0.588	0.842	0.714	0.657	0.909	0.432
DT	0.682	0.842	0.779	0.730	0.909	0.533
KNN	0.769	0.857	0.847	0.791	0.943	0.545
GNB	0.806	0.857	0.870	0.819	0.943	0.600
SVM	0.857	0.842	0.882	0.852	0.909	0.762
LR	0.851	0.857	0.889	0.853	0.930	0.720
RF	0.909	0.842	0.909	0.885	0.909	0.842

**Table 11:** Statistical analysis of the results of the classification results.

D1	DBERDTO + RF	BER + RF	DTO + RF	PSO + RF	WAO + RF	GWO + RF	FA + RF
Number of values	10	10	10	10	10	10	10
Minimum	0.986	0.951	0.950	0.933	0.928	0.927	0.923
25% Percentile	0.986	0.961	0.959	0.943	0.938	0.937	0.927
Median	0.986	0.961	0.959	0.943	0.938	0.937	0.927
75% Percentile	0.986	0.961	0.959	0.943	0.941	0.937	0.927
Maximum	0.992	0.961	0.960	0.953	0.958	0.940	0.936
Range	0.006	0.010	0.010	0.020	0.030	0.012	0.013
10% Percentile	0.986	0.952	0.951	0.934	0.929	0.928	0.923
90% Percentile	0.991	0.961	0.960	0.952	0.957	0.940	0.935
Actual confidence level	97.85%	97.85%	97.85%	97.85%	97.85%	97.85%	97.85%
Lower confidence limit	0.986	0.959	0.959	0.943	0.938	0.937	0.927
Upper confidence limit	0.986	0.961	0.959	0.943	0.948	0.937	0.927
Mean	0.986	0.960	0.958	0.943	0.940	0.937	0.927
Std. Deviation	0.002	0.003	0.003	0.005	0.008	0.003	0.003
Std. Error of Mean	0.001	0.001	0.001	0.001	0.002	0.001	0.001
Lower 95% CI of mean	0.985	0.958	0.956	0.940	0.935	0.934	0.925
Upper 95% CI of mean	0.988	0.962	0.960	0.947	0.946	0.939	0.929
Coefficient of variation	0.1911%	0.3289%	0.3028%	0.4997%	0.8391%	0.3562%	0.3518%
Geometric mean	0.986	0.960	0.958	0.943	0.940	0.937	0.927
Geometric SD factor	1.002	1.003	1.003	1.005	1.008	1.004	1.004
Lower 95% CI of geo. mean	0.985	0.958	0.956	0.940	0.935	0.934	0.925
Upper 95% CI of geo. mean	0.988	0.962	0.960	0.947	0.946	0.939	0.929
Harmonic mean	0.986	0.960	0.958	0.943	0.940	0.937	0.927
Lower 95% CI of harm. mean	0.985	0.958	0.956	0.940	0.935	0.934	0.925
Upper 95% CI of harm. mean	0.988	0.962	0.960	0.947	0.946	0.939	0.929
Quadratic mean	0.986	0.960	0.958	0.943	0.940	0.937	0.927
Lower 95% CI of quad. mean	0.985	0.958	0.956	0.940	0.934	0.934	0.925
Upper 95% CI of quad. mean	0.988	0.962	0.960	0.947	0.946	0.939	0.929
Skewness	3.162	−2.939	−3.090	0.000	1.290	−2.819	2.223
Kurtosis	10.000	8.818	9.695	4.500	2.985	8.705	7.021
Sum	9.862	9.600	9.581	9.433	9.401	9.365	9.270

**Table 12:** ANOVA test applied to the results of the optimized RF classifier.

D1	SS	DF	MS	F (DFn, DFd)	*p*-Value
Treatment	0.02327	6	0.003878	F (6, 63) = 211.8	*p* < 0.0001
Residual	0.00115	63	0.000018		
Total	0.02442	69			

**Table 13:** Wilcoxon test applied to the results of the optimized RF classifier.

D1	DBERDTO + RF	BER + RF	DTO + RF	PSO + RF	WAO + RF	GWO + RF	FA + RF
Number of values	10	10	10	10	10	10	10
Actual median	0.9856	0.9612	0.9589	0.9433	0.9381	0.9373	0.9265
Theoretical median	0	0	0	0	0	0	0
Sum of negative ranks	0	0	0	0	0	0	0
Sum of signed ranks (W)	55	55	55	55	55	55	55
Sum of positive ranks	55	55	55	55	55	55	55
P value (two tailed)	0.002	0.002	0.002	0.002	0.002	0.002	0.002
Exact or estimate?	Exact	Exact	Exact	Exact	Exact	Exact	Exact
Discrepancy	0.9856	0.9612	0.9589	0.9433	0.9381	0.9373	0.9265
Significant (alpha = 0.05)?	Yes	Yes	Yes	Yes	Yes	Yes	Yes

**Figure A1:**
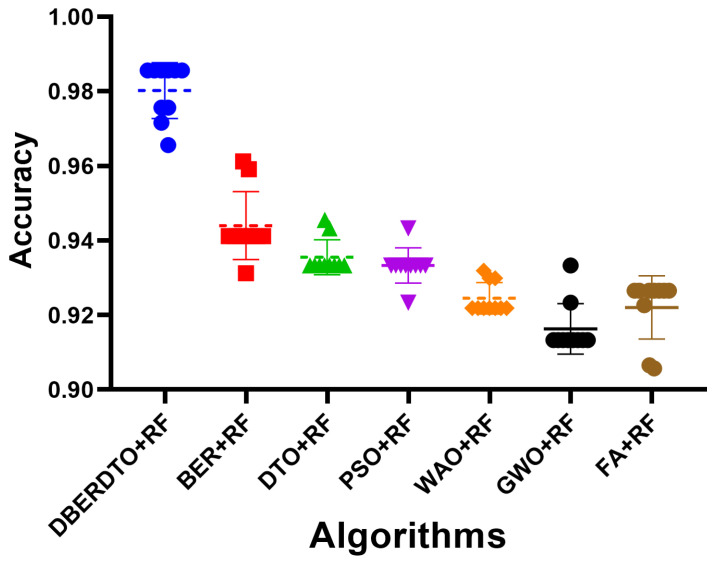
The classification accuracy of diabetes in dataset D2 after feature selection using the proposed approach.

**Figure A2:**
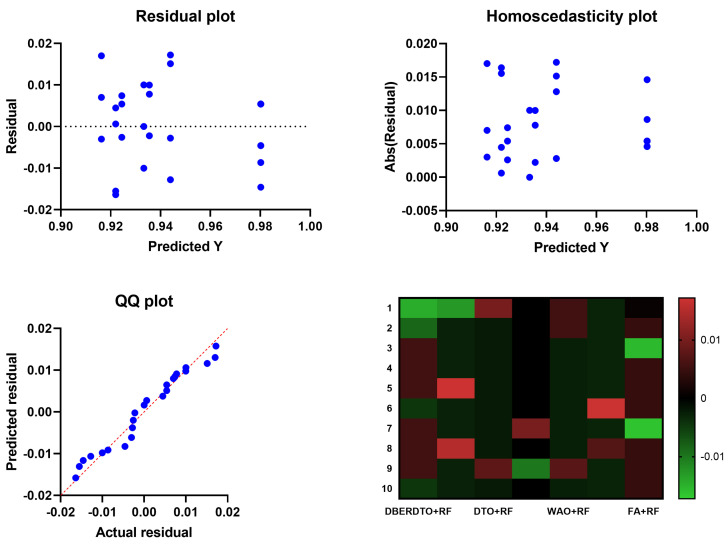
Visualizing ANOVA test results applied to the optimized RF classifier outcomes.

**Figure A3:**
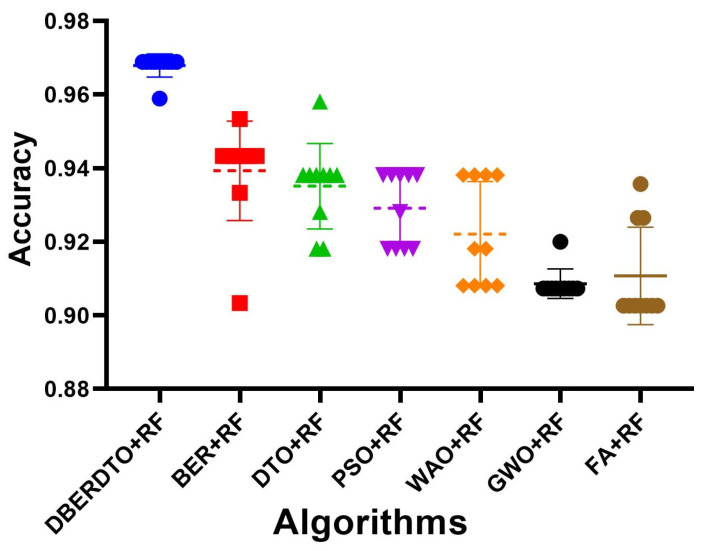
The classification accuracy of diabetes in dataset D3 after feature selection using the proposed approach.

**Figure A4:**
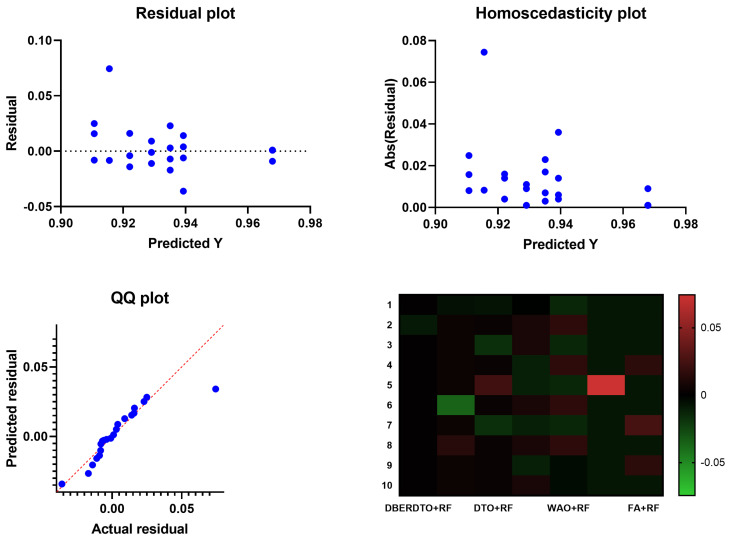
Visualizing ANOVA test results when applied to the results of the optimized RF classifier.

**Figure A5:**
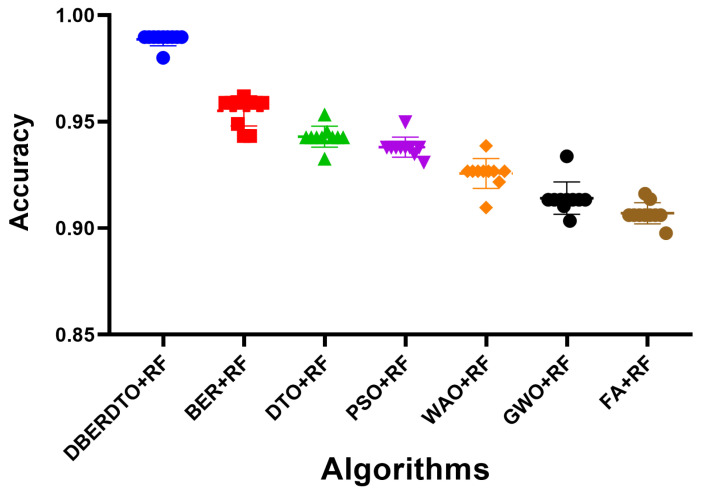
The classification accuracy of diabetes in dataset D4 after feature selection using the proposed approach.

**Figure A6:**
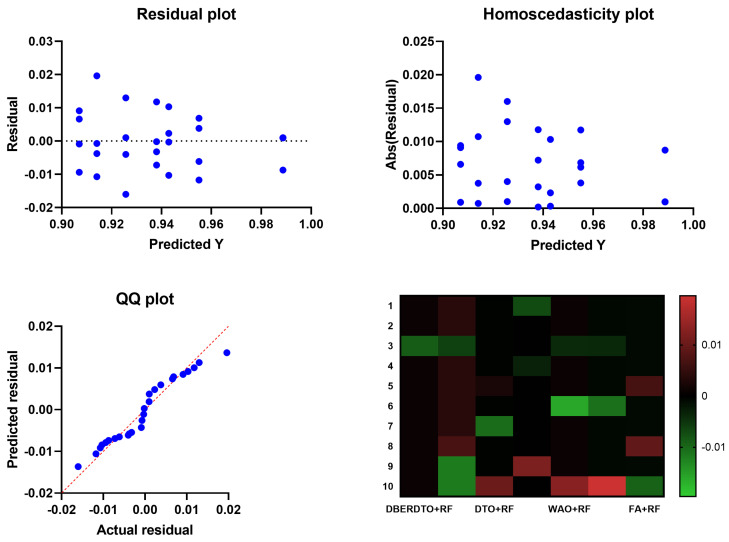
Visualizing ANOVA test results when applied to the results of the optimized RF classifier.

## Data Availability

Dataset (D1): https://www.kaggle.com/datasets/mathchi/diabetes-data-set, Dataset (D2): https://www.kaggle.com/datasets/akshaydattatraykhare/diabetes-dataset, Dataset (D3): https://data.mendeley.com/datasets/wj9rwkp9c2/1, Dataset (D4): https://www.kaggle.com/datasets/uciml/pima-indians-diabetes-database (accessed on 29 April 2023).

## Appendix A

In this appendix, the proposed feature selection method is applied to three other datasets to prove its generalization and superiority in the classification of diabetes. The first dataset, denoted by D2, is originally from the National Institute of Diabetes and Digestive and Kidney Diseases [50]. The second dataset, denoted by D3, is collected from the Iraqi society, as the data were acquired from the laboratory of Medical City Hospital [51]. The third dataset, denoted by D4, is originally from the National Institute of Diabetes and Digestive and Kidney Diseases [52]. In the next sections, the proposed approach is evaluated in terms of the adopted criteria.

### Appendix A.1. The Achieved Results Using Dataset D2

The proposed feature selection method bDBERDTO has been used for selecting the significant set of features in dataset D2 and the results are presented in Table A1. The results of the feature selection analysis using the bDBERDTO method on dataset D2, along with various other methods, including bBER, bDTO, bPSO, bWAO, bGWO, and bFA, reveal interesting insights. The bDBERDTO method demonstrates its effectiveness by achieving a notably low average error of 0.819, indicating improved accuracy in selecting relevant features. Moreover, bDBERDTO stands out by selecting a comparatively smaller subset of features, as evidenced by its average select size of 0.772. This suggests a more efficient and concise feature selection process. When considering the average fitness value, bDBERDTO attains a respectable score of 0.882, indicating strong overall performance. Notably, the best fitness value obtained by bDBERDTO is 0.784, signifying its ability to identify the most optimal subset of features within the dataset. Even in the worst-case scenarios, bDBERDTO maintains competitive performance with the worst fitness value of 0.882. The standard deviation of fitness, which is 0.704 for bDBERDTO, indicates consistent and stable results across different iterations. These results collectively showcase the bDBERDTO method’s efficacy in achieving low average error, selecting an optimal subset of features, and maintaining competitive fitness values.

**Table A1:** Evaluation of the results of the proposed feature selection method when applied to dataset D2.

D2	bDBERDTO	bBER	bDTO	bPSO	bWAO	bGWO	bFA
Avg. error	0.819	0.836	0.850	0.870	0.870	0.856	0.868
Avg. Select size	0.772	0.972	0.914	0.972	1.135	0.894	1.006
Avg. Fitness	0.882	0.898	0.910	0.897	0.904	0.904	0.949
Best Fitness	0.784	0.819	0.813	0.877	0.869	0.882	0.867
Worst Fitness	0.882	0.885	0.928	0.945	0.945	0.958	0.965
Std. Fitness	0.704	0.709	0.711	0.708	0.711	0.710	0.745

The classification results of the diabetes dataset D2, before applying feature selection, provide valuable insights into the performance of different classifiers as presented in Table A2. Among the classifiers evaluated, Random Forest demonstrates the highest positive predictive value (PPV) of 0.830, indicating its ability to identify positive instances correctly. Additionally, Random Forest exhibits a high negative predictive value (NPV) of 0.838, indicating its proficiency in accurately identifying negative instances. The F-score, which considers both precision and recall, is relatively high for Gaussian NB, reaching 0.686, suggesting its balanced performance. Accuracy, a measure of overall correctness, is highest for SVC, achieving a value of 0.770. Sensitivity, or true positive rate, is notably high for K-Neighbors, indicating its ability to classify positive instances correctly. On the other hand, specificity, or true negative rate, is highest for SGD, demonstrating its capability to classify negative instances accurately. Overall, these classification results provide a comprehensive overview of the performance of different classifiers on the diabetes dataset D2, serving as a baseline for evaluating the impact of feature selection techniques on their performance.

**Table A2:** Evaluation of the classifier results before applying feature selection in terms of dataset D2.

D2	PPV	NPV	F-Score	Accuracy	Sensitivity	Specificity
SGD	0.720	0.494	0.435	0.526	0.375	0.766
Decision Tree	0.746	0.721	0.571	0.671	0.697	0.630
K-Neighbors	0.716	0.753	0.617	0.684	0.794	0.511
Gaussian NB	0.803	0.798	0.686	0.757	0.794	0.698
SVC	0.751	0.828	0.827	0.770	0.923	0.528
Logistic Regression	0.790	0.822	0.750	0.776	0.858	0.647
Random Forest	0.830	0.838	0.758	0.803	0.848	0.732

After applying feature selection to the diabetes dataset D2, the classification results demonstrate improvements in the performance of the evaluated classifiers. The classification results are shown in Table A3 and in Figure A1. SGD, which initially had a relatively low PPV, shows an increase to 0.596, indicating better identification of positive instances. Notably, Decision Tree substantially enhances its F-score, reaching 0.789, suggesting improved precision and recall balance. K-Neighbors demonstrate remarkable progress in multiple performance metrics, including an elevated PPV of 0.779, NPV of 0.868, and F-score of 0.859, indicating its ability to classify both positive and negative instances accurately. Gaussian NB showcases significant improvements in all metrics, with high PPV, NPV, and F-score values of 0.817, 0.868, and 0.881, respectively. SVC demonstrates enhanced performance in terms of PPV, NPV, and F-score, indicating improved accuracy and reliability. Logistic Regression exhibits a high F-score of 0.901, emphasizing its ability to balance precision and recall. Finally, Random Forest maintains its strong performance, achieving a high PPV of 0.921 and an F-score of 0.921, highlighting its effectiveness in accurately classifying instances. Overall, the feature selection process enhances the performance of the classifiers, resulting in improved accuracy and precision in classifying instances in the diabetes dataset D2.

**Table A3:** Evaluation of the classifier results after applying feature selection using the proposed bDBERDTO in terms of dataset D2.

D2	PPV	NPV	F-Score	Accuracy	Sensitivity	Specificity
SGD	0.596	0.853	0.724	0.666	0.921	0.438
Decision Tree	0.691	0.853	0.789	0.740	0.921	0.540
K-Neighbors	0.779	0.868	0.859	0.801	0.956	0.553
Gaussian NB	0.817	0.868	0.881	0.830	0.956	0.608
SVC	0.868	0.853	0.894	0.863	0.921	0.772
Logistic Regression	0.862	0.868	0.901	0.864	0.942	0.729
Random Forest	0.921	0.853	0.921	0.896	0.921	0.853

The ANOVA test was conducted on the classification results of the optimized Random Forest classifier for the diabetes dataset D2 and the results are presented in Table A4 and in Figure A2. The test evaluated the sources of variation between columns (treatments) and within columns (residuals) to determine their significance in explaining the overall variation. The treatment analysis showed a sum of squares (SS) of 0.027, degrees of freedom (DF) of 6, and mean square (MS) of 0.0046. The F-statistic (6, 63) = 100.3 indicated a highly significant difference between the treatment means. The corresponding *p*-value was found to be less than 0.0001, further confirming the statistical significance of the observed differences. The residual analysis, representing the unexplained variation within columns, yielded an SS of 0.003, DF of 63, and MS of 0.00005. The total variation accounted for a cumulative SS of 0.031 and a DF of 69. These results indicate that the optimized Random Forest classifier for the diabetes dataset D2 exhibits significant differences in performance across the evaluated treatments, highlighting the impact of feature selection on the classification outcomes.

**Table A4:** ANOVA test applied to the results of the optimized RF classifier in terms of the dataset D2.

D2	SS	DF	MS	F (DFn, DFd)	*p*-Value
Treatment (between columns)	0.027	6	0.0046	F (6, 63) = 100.3	*p* < 0.0001
Residual (within columns)	0.003	63	0.00005		
Total	0.031	69			

The Wilcoxon test was performed on the classification results of the optimized Random Forest classifier for the diabetes dataset D2, comparing different feature selection methods combined with Random Forest (RF). The results of this test are presented in Table A5. The test aimed to determine if there were significant differences in the performance of these combinations compared to the theoretical median of 0. The median values for DBERDTO + RF, BER + RF, DTO + RF, PSO + RF, WAO + RF, GWO + RF, and FA + RF were found to be 0.986, 0.941, 0.933, 0.933, 0.922, 0.913, and 0.926, respectively. The Wilcoxon test statistics showed that the sum of signed ranks for each combination was 55, indicating no significant deviations from the median. The sum of positive ranks and the sum of negative ranks were both 55, suggesting a balanced distribution. The resulting *p*-values for all combinations were calculated as 0.002, less than the significance level of 0.05. Therefore, it can be concluded that there is strong evidence to reject the null hypothesis and assert that these feature selection methods combined with Random Forest significantly outperformed the theoretical median of 0 in the classification of the diabetes dataset D2. In addition, the statistical results presented in Table A6 demonstrate an in-depth investigation of the statistical significance of the proposed methodology.

**Table A5:** Wilcoxon test applied to the results of the optimized RF classifier in terms of dataset D2.

D2	DBERDTO + RF	BER + RF	DTO + RF	PSO + RF	WAO + RF	GWO + RF	FA + RF
Theoretical median	0	0	0	0	0	0	0
Median	0.986	0.941	0.933	0.933	0.922	0.913	0.927
Number of values	10	10	10	10	10	10	10
Sum of signed ranks	55	55	55	55	55	55	55
Sum of +ve ranks	55	55	55	55	55	55	55
Sum of −ve ranks	0	0	0	0	0	0	0
*p*-value	0.002	0.002	0.002	0.002	0.002	0.002	0.002
Discrepancy	0.986	0.941	0.933	0.933	0.922	0.913	0.927

**Table A6:** Statistical analysis of the classification results generated by the optimized RF classifier applied to dataset D2.

D2	DBERDTO + RF	BER + RF	DTO + RF	PSO + RF	WAO + RF	GWO + RF	FA + RF
Number of values	10.000	10.000	10.000	10.000	10.000	10.000	10.000
Minimum	0.966	0.931	0.933	0.923	0.922	0.913	0.906
25% Percentile	0.975	0.941	0.933	0.933	0.922	0.913	0.919
Median	0.986	0.941	0.933	0.933	0.922	0.913	0.927
75% Percentile	0.986	0.946	0.936	0.933	0.930	0.916	0.927
Maximum	0.986	0.961	0.946	0.943	0.932	0.933	0.927
Range	0.020	0.030	0.012	0.020	0.010	0.020	0.021
10% Percentile	0.966	0.932	0.933	0.924	0.922	0.913	0.906
90% Percentile	0.986	0.961	0.945	0.942	0.932	0.932	0.927
Actual confidence level	97.85%	97.85%	97.85%	97.85%	97.85%	97.85%	97.85%
Lower confidence limit	0.972	0.941	0.933	0.933	0.922	0.913	0.907
Upper confidence limit	0.986	0.959	0.943	0.933	0.930	0.923	0.927
Mean	0.980	0.944	0.936	0.933	0.925	0.916	0.922
Std. Deviation	0.007	0.009	0.005	0.005	0.004	0.007	0.008
Std. Error of Mean	0.002	0.003	0.001	0.001	0.001	0.002	0.003
Lower 95% CI of mean	0.975	0.938	0.932	0.930	0.922	0.912	0.916
Upper 95% CI of mean	0.986	0.951	0.939	0.937	0.928	0.921	0.928
Coefficient of variation	0.7643%	0.9626%	0.5033%	0.5051%	0.4566%	0.7366%	0.9215%
Geometric mean	0.980	0.944	0.936	0.933	0.925	0.916	0.922
Geometric SD factor	1.008	1.010	1.005	1.005	1.005	1.007	1.009
Lower 95% CI of geo. mean	0.975	0.938	0.932	0.930	0.922	0.912	0.916
Upper 95% CI of geo. mean	0.986	0.950	0.939	0.937	0.928	0.921	0.928
Harmonic mean	0.980	0.944	0.936	0.933	0.925	0.916	0.922
Lower 95% CI of harm. mean	0.975	0.938	0.932	0.930	0.922	0.912	0.916
Upper 95% CI of harm. mean	0.986	0.950	0.939	0.937	0.928	0.921	0.928
Quadratic mean	0.980	0.944	0.936	0.933	0.925	0.916	0.922
Lower 95% CI of quad. mean	0.975	0.938	0.932	0.930	0.922	0.911	0.916
Upper 95% CI of quad. mean	0.986	0.951	0.939	0.937	0.928	0.921	0.928
Skewness	−0.991	1.168	1.832	0.000	1.102	2.277	−1.702
Kurtosis	−0.392	1.015	1.777	4.500	−0.891	4.765	1.228
Sum	9.802	9.440	9.355	9.333	9.245	9.163	9.220

### Appendix A.2. The Achieved Results Using Dataset D3

Similarly, the proposed feature selection method is applied to the features in the third dataset denoted by D3. The evaluation results of the selected features are presented in Table A7. The feature selection evaluation using the proposed bDBERDTO method for the diabetes dataset D3 provides valuable insights into its performance. The average error rates obtained using various feature selection algorithms range from 0.738 to 0.787, with bDBERDTO achieving an average error rate of 0.738. The average select size represents the number of features selected, ranging from 0.691 to 1.054, with bWAO having the largest average select size of 1.054. The average fitness scores, which measure the quality of the selected features, range from 0.801 to 0.868. The best fitness scores indicate the highest achieved fitness for each algorithm, ranging from 0.703 to 0.786. Conversely, the worst fitness scores range from 0.801 to 0.884. The standard deviation of fitness scores ranges from 0.623 to 0.664, indicating the degree of variability in the performance of the algorithms. Overall, the bDBERDTO method demonstrates competitive performance across various evaluation metrics, showcasing its potential as an effective feature selection technique for the diabetes dataset D3.

**Table A7:** Evaluation of the results of the proposed feature selection method when applied to dataset D3.

D3	bDBERDTO	bBER	bDTO	bPSO	bWAO	bGWO	bFA
Avg. error	0.738	0.755	0.769	0.789	0.789	0.775	0.787
Avg. Select size	0.691	0.891	0.833	0.891	1.054	0.814	0.925
Avg. Fitness	0.801	0.817	0.829	0.816	0.824	0.823	0.868
Best Fitness	0.703	0.738	0.732	0.796	0.788	0.801	0.786
Worst Fitness	0.801	0.805	0.847	0.864	0.864	0.877	0.884
Std. Fitness	0.623	0.628	0.630	0.628	0.630	0.629	0.664

The evaluation of classifier results for the dataset D3 prior to applying feature selection provides valuable insights into their performance as presented in Table A8. The SGD classifier achieved an accuracy of 0.480, indicating that it correctly classified approximately 48% of the instances. The Decision Tree classifier performed slightly better, with an accuracy of 0.612. The K-Neighbors classifier achieved an accuracy of 0.624, demonstrating its ability to classify instances with higher accuracy. The Gaussian NB classifier exhibited improved performance, with an accuracy of 0.690, indicating its effectiveness in handling the features of the dataset. The SVC classifier achieved an accuracy of 0.702, demonstrating its ability to classify instances with higher accuracy correctly. The Logistic Regression classifier achieved an accuracy of 0.708, while the Random Forest classifier achieved an accuracy of 0.732, showcasing their ability to classify instances more accurately than previous classifiers. Overall, the evaluation of classifier results for dataset D3 highlights the varying performance levels of different classifiers and provides valuable insights for further analysis and improvement.

**Table A8:** Evaluation of the classifier results before applying feature selection in terms of dataset D3.

D3	PPV	NPV	F-Score	Accuracy	Sensitivity	Specificity
SGD	0.656	0.450	0.397	0.480	0.342	0.698
Decision Tree	0.680	0.657	0.520	0.612	0.636	0.574
K-Neighbors	0.653	0.687	0.562	0.624	0.724	0.465
Gaussian NB	0.732	0.728	0.625	0.690	0.724	0.636
SVC	0.685	0.755	0.754	0.702	0.841	0.481
Logistic Regression	0.720	0.750	0.684	0.708	0.783	0.590
Random Forest	0.756	0.764	0.691	0.732	0.773	0.667

The evaluation of classifier results after applying feature selection using the proposed bDBERDTO method on dataset D3 provides valuable insights into the performance of the classifiers as presented in Table A9 and in Figure A3. The SGD classifier achieved a precision of 0.529, indicating its ability to classify positive instances correctly, and a sensitivity of 0.818, suggesting its effectiveness in identifying true positive instances. However, its specificity was lower at 0.389, indicating a higher rate of false positives. The Decision Tree classifier showed improvements with a precision of 0.614, sensitivity of 0.818, and specificity of 0.480, indicating a more balanced performance. The K-Neighbors classifier demonstrated even better performance with a precision of 0.692, sensitivity of 0.849, and specificity of 0.491. The Gaussian NB classifier improved the results with a precision of 0.726, sensitivity of 0.849, and specificity of 0.540. The SVC classifier achieved high precision (0.771) and specificity (0.686), accurately classifying instances in both positive and negative classes. The Logistic Regression classifier achieved a precision of 0.766, sensitivity of 0.837, and specificity of 0.648, indicating its balanced performance. Finally, the Random Forest classifier demonstrated excellent performance across all metrics, with precision, sensitivity, and specificity of 0.889. Overall, the evaluation of classifier results after applying feature selection using the bDBERDTO method on dataset D3 showcases the improvement in classifier performance, particularly in terms of precision, sensitivity, and specificity, leading to more accurate and reliable classifications.

**Table A9:** Evaluation of the classifier results after applying feature selection using the proposed bDBERDTO in terms of dataset D3.

D3	PPV	NPV	F-Score	Accuracy	Sensitivity	Specificity
SGD	0.529	0.758	0.643	0.591	0.818	0.389
Decision Tree	0.614	0.758	0.701	0.657	0.818	0.480
K-Neighbors	0.692	0.771	0.763	0.712	0.849	0.491
Gaussian NB	0.726	0.771	0.783	0.737	0.849	0.540
SVC	0.771	0.758	0.794	0.767	0.818	0.686
Logistic Regression	0.766	0.771	0.800	0.768	0.837	0.648
Random Forest	0.889	0.823	0.889	0.865	0.889	0.823

The ANOVA test was conducted on the results of the classification using the optimized random forest classifier on dataset D3 as presented in Table A10 and in Figure A4. The test aimed to analyze the variation between the different treatments (classifier methods) and within the columns (residuals). The results showed that the treatment (between columns) accounted for significant variation, as indicated by the relatively high sum of squares (SS) value of 0.022. With 6 degrees of freedom (DF), the mean squares (MS) value was calculated as 0.0036. The F-value, obtained by dividing the treatment MS by the residual MS, was determined to be F (6, 63) = 17.07, indicating a significant difference between the treatment groups. The *p*-value associated with the F-test was less than 0.0001, further confirming the statistical significance of the results. In contrast, the residual (within columns) accounted for a lower variation, as indicated by the SS value of 0.013 and 63 degrees of freedom. The total sum of squares was determined to be 0.035, representing the overall variation in the dataset. These ANOVA test results provide evidence that the different classifier methods significantly impacted the classification results, underscoring the importance of selecting an optimized random forest classifier for accurate predictions on the D3 dataset.

**Table A10:** ANOVA test applied to the results of the optimized RF classifier in terms of dataset D3.

D3	SS	DF	MS	F (DFn, DFd)	*p*-Value
Treatment (between columns)	0.022	6	0.0036	F (6, 63) = 17.07	*p* < 0.0001
Residual (within columns)	0.013	63	0.0002		
Total	0.035	69			

The Wilcoxon test was performed on the results of the optimized random forest classifier on the D3 dataset to assess the statistical significance of the differences between different feature selection methods as presented in Table A11. The test was conducted by comparing the performance of the feature selection methods, including DBERDTO + RF, BER + RF, DTO + RF, PSO + RF, WAO + RF, GWO + RF, and FA + RF. The theoretical median for all methods was set to 0. The actual medians were calculated as 0.9689, 0.9433, 0.9381, 0.9331, 0.9181, 0.9073, and 0.9027 for each method. The Wilcoxon test was applied to the dataset with 10 values, resulting in a sum of signed ranks of 55. All ranks were positive, with a sum of positive ranks also equal to 55. The sum of negative ranks was 0, indicating no negative differences. The *p*-value for the test was determined to be 0.002, suggesting a statistically significant difference between the feature selection methods. The discrepancy values matched the actual medians for each method. Overall, these Wilcoxon test results provide evidence of significant differences among the feature selection methods when combined with the optimized random forest classifier on the D3 dataset.

The statistical analysis of the results achieved by the optimized random forest classifier on the D3 dataset provides insights into the performance of various feature selection methods as presented in Table A12. The analysis reveals the minimum, median, range, and percentile values for each method, indicating variations in performance. Mean values, standard deviation, and confidence intervals are computed, offering a comprehensive understanding of the spread and variation of the results. Other measures such as coefficient of variation, geometric mean, harmonic mean, quadratic mean, skewness, kurtosis, and sum provide additional information about the data distribution and overall classifier performance. This analysis aids in informed decision-making regarding selecting the most effective feature selection approach.

**Table A11:** Wilcoxon test applied to the results of the optimized RF classifier in terms of dataset D3.

D3	DBERDTO + RF	BER + RF	DTO + RF	PSO + RF	WAO + RF	GWO + RF	FA + RF
Theoretical median	0	0	0	0	0	0	0
Actual median	0.969	0.943	0.938	0.933	0.918	0.907	0.903
Number of values	10	10	10	10	10	10	10
Sum of signed ranks	55	55	55	55	55	55	55
Sum of +ve ranks	55	55	55	55	55	55	55
Sum of −ve ranks	0	0	0	0	0	0	0
*p*-value	0.002	0.002	0.002	0.002	0.002	0.002	0.002
Discrepancy	0.969	0.943	0.938	0.933	0.918	0.907	0.903

**Table A12:** Statistical analysis of the classification results generated by the optimized RF classifier applied to dataset D3.

D3	DBERDTO + RF	BER + RF	DTO + RF	PSO + RF	WAO + RF	GWO + RF	FA + RF
Number of values	10	10	10	10	10	10	10
Minimum	0.9589	0.9033	0.9181	0.9181	0.9081	0.9073	0.9027
25% Percentile	0.9689	0.9408	0.9256	0.9181	0.9081	0.9073	0.9027
Median	0.9689	0.9433	0.9381	0.9331	0.9181	0.9073	0.9027
75% Percentile	0.9689	0.9433	0.9381	0.9381	0.9381	0.9073	0.9265
Maximum	0.9689	0.9533	0.9581	0.9381	0.9381	0.92	0.9357
Range	0.010	0.050	0.040	0.020	0.030	0.01267	0.033
10% Percentile	0.9599	0.9063	0.9181	0.9181	0.9081	0.9073	0.9027
90% Percentile	0.9689	0.9523	0.9561	0.9381	0.9381	0.9187	0.9347
Actual confidence level	97.85%	97.85%	97.85%	97.85%	97.85%	97.85%	97.85%
Lower confidence limit	0.9689	0.9333	0.9181	0.9181	0.9081	0.9073	0.9027
Upper confidence limit	0.9689	0.9433	0.9381	0.9381	0.9381	0.9073	0.9265
Mean	0.9679	0.9393	0.9351	0.9291	0.9221	0.9086	0.9107
Std. Deviation	0.0032	0.0135	0.0116	0.0099	0.0143	0.00401	0.0132
Std. Error of Mean	0.001	0.0043	0.0037	0.0032	0.0045	0.0013	0.0042
Lower 95% CI of mean	0.9656	0.9296	0.9268	0.922	0.9119	0.9057	0.9013
Upper 95% CI of mean	0.9702	0.949	0.9434	0.9362	0.9323	0.9114	0.9202
Coefficient of variation	0.3267%	1.437%	1.240%	1.070%	1.551%	0.4411%	1.453%
Geometric mean	0.9679	0.9392	0.935	0.9291	0.922	0.9086	0.9106
Geometric SD factor	1.003	1.015	1.012	1.011	1.016	1.004	1.015
Lower 95% CI of geo. mean	0.9656	0.9295	0.9268	0.922	0.9118	0.9057	0.9013
Upper 95% CI of geo. mean	0.9702	0.9491	0.9434	0.9362	0.9323	0.9114	0.9201
Harmonic mean	0.9679	0.9391	0.935	0.929	0.9219	0.9086	0.9105
Lower 95% CI of harm. mean	0.9656	0.9293	0.9268	0.9219	0.9118	0.9057	0.9013
Upper 95% CI of harm. mean	0.9702	0.9492	0.9433	0.9362	0.9322	0.9114	0.92
Quadratic mean	0.9679	0.9394	0.9352	0.9291	0.9222	0.9086	0.9108
Lower 95% CI of quad. mean	0.9656	0.9298	0.9268	0.922	0.9119	0.9057	0.9012
Upper 95% CI of quad. mean	0.9702	0.9489	0.9434	0.9362	0.9324	0.9115	0.9203
Skewness	−3.162	−2.466	0.1924	−0.2373	0.2509	3.162	1.18
Kurtosis	10.0	6.974	1.092	−2.30	−2.165	10.0	−0.4931
Sum	9.679	9.393	9.351	9.291	9.221	9.086	9.107

### Appendix A.3. The Achieved Results Using Dataset D4

Finally, the proposed approach is evaluated in terms of a dataset addressed by several researchers in the literature [53,54,55,56]. This dataset is originally from the National Institute of Diabetes and Digestive and Kidney Diseases [52]. The proposed approach is used to select the best set of features to boost classification accuracy. The results are evaluated using the adopted metrics, and various statistical tests were conducted to study the statistical difference and significance of the proposed approach. Feature selection results in terms of dataset D4 are presented in Table A13. As presented in this table, the proposed bBERDTO achieves the best results when compared to the other optimization methods.

**Table A13:** Evaluation of the results of the proposed feature selection method when applied to dataset D4.

D4	bDBERDTO	bBER	bDTO	bPSO	bWAO	bGWO	bFA
Average error	0.718	0.735	0.749	0.769	0.769	0.755	0.768
Average Select size	0.671	0.871	0.813	0.871	1.034	0.794	0.905
Average Fitness	0.781	0.798	0.809	0.796	0.804	0.804	0.848
Best Fitness	0.683	0.718	0.712	0.776	0.768	0.781	0.767
Worst Fitness	0.782	0.785	0.827	0.844	0.844	0.858	0.864
Std Fitness	0.604	0.608	0.610	0.608	0.610	0.609	0.645

To determine the best classification model for this dataset, a set of experiments is conducted to assess seven machine learning models, and the results before feature selection and after feature selection are presented in Table A14 and Table A15, respectively. As presented in these tables, the Random Forest classifier is the best classifier, which achieves the best results before and after feature selection.

**Table A14:** Evaluation of the classifier results before applying feature selection in terms of dataset D4.

D4	PPV	NPV	F-Score	Accuracy	Sensitivity	Specificity
SGD	0.713	0.489	0.431	0.521	0.372	0.759
Decision Tree	0.739	0.714	0.565	0.665	0.691	0.624
K-Neighbors	0.709	0.746	0.611	0.678	0.786	0.506
Gaussian NB	0.795	0.791	0.679	0.750	0.786	0.691
SVC	0.744	0.820	0.819	0.763	0.914	0.523
Logistic Regression	0.782	0.815	0.743	0.769	0.850	0.641
Random Forest	0.822	0.831	0.751	0.795	0.840	0.725

**Table A15:** Evaluation of the classifier results after applying feature selection in terms of dataset D4.

D4	PPV	NPV	F-Score	Accuracy	Sensitivity	Specificity
SGD Classifier	0.576	0.824	0.699	0.643	0.890	0.423
Decision Tree Classifier	0.667	0.824	0.763	0.715	0.890	0.522
K-Neighbors Classifier	0.753	0.839	0.830	0.774	0.923	0.534
Gaussian NB	0.789	0.839	0.851	0.802	0.923	0.587
SVC	0.839	0.824	0.864	0.834	0.890	0.746
Logistic Regression	0.833	0.839	0.870	0.835	0.911	0.705
Random Forest Classifier	0.890	0.824	0.890	0.866	0.890	0.824

The accuracy of the optimized random forest classifier fed with the best set of features is depicted in the plot shown in Figure A5. In this plot, the average accuracy achieved by the proposed approach is 98.9%. This result is superior to that of the other competing approaches proposed by the authors in Refs. [53,54,55,56].

On the other hand, a set of statistical tests has been conducted to study the statistical difference and significance of the proposed approach. This set is composed of the ANOVA, Wilcoxon, and statistical analysis. The results of these tests are presented in Table A16, Table A17, and Table A18, respectively. The results presented in these tables confirm the expected outcomes of the proposed approach. In addition, the plots shown in Figure A6 depict the visual representation of the ANOVA test results in terms of the Residual, Homoscedasticity, QQ, and Heatmap plots. These plots emphasize the effectiveness and statistical difference of the proposed approach when compared to the other approaches based on different optimization methods.

**Table A16:** ANOVA test applied to the results of the optimized RF classifier in terms of dataset D4.

D4	SS	DF	MS	F (DFn, DFd)	*p*-Value
Treatment (between columns)	0.046	6	0.007603	F (6, 63) = 223.9	*p* < 0.0001
Residual (within columns)	0.002	63	0.00003395		
Total	0.048	69			

**Table A17:** Wilcoxon test applied to the results of the optimized RF classifier in terms of dataset D4.

D4	DBERDTO + RF	BER + RF	DTO + RF	PSO + RF	WAO + RF	GWO + RF	FA + RF
Theoretical median	0	0	0	0	0	0	0
Actual median	0.990	0.959	0.943	0.938	0.927	0.913	0.906
Number of values	10	10	10	10	10	10	10
Sum of signed ranks (W)	55	55	55	55	55	55	55
Sum of positive ranks	55	55	55	55	55	55	55
Sum of negative ranks	0	0	0	0	0	0	0
P value (two tailed)	0.002	0.002	0.002	0.002	0.002	0.002	0.002
Exact or estimate?	Exact	Exact	Exact	Exact	Exact	Exact	Exact
Discrepancy	0.990	0.959	0.943	0.938	0.927	0.913	0.906

**Table A18:** Statistical analysis of the classification results generated by the optimized RF classifier applied to dataset D4.

D4	DBERDTO + RF	BER + RF	DTO + RF	PSO + RF	WAO + RF	GWO + RF	FA + RF
Number of values	10	10	10	10	10	10	10
Minimum	0.980	0.943	0.933	0.931	0.910	0.903	0.898
25% Percentile	0.990	0.948	0.943	0.937	0.925	0.913	0.906
Median	0.990	0.959	0.943	0.938	0.927	0.913	0.906
75% Percentile	0.990	0.959	0.943	0.938	0.927	0.913	0.908
Maximum	0.990	0.962	0.953	0.950	0.939	0.934	0.916
Range	0.010	0.019	0.021	0.019	0.029	0.030	0.019
10% Percentile	0.981	0.943	0.934	0.931	0.911	0.904	0.899
90% Percentile	0.990	0.962	0.953	0.949	0.938	0.932	0.916
Actual confidence level	97.85%	97.85%	97.85%	97.85%	97.85%	97.85%	97.85%
Lower confidence limit	0.990	0.943	0.943	0.935	0.922	0.910	0.906
Upper confidence limit	0.990	0.959	0.945	0.938	0.927	0.913	0.914
Mean	0.989	0.955	0.943	0.938	0.926	0.914	0.907
Std. Deviation	0.003	0.007	0.005	0.005	0.007	0.008	0.005
Std. Error of Mean	0.001	0.002	0.002	0.001	0.002	0.002	0.002
Lower 95% CI of mean	0.987	0.950	0.939	0.935	0.921	0.909	0.904
Upper 95% CI of mean	0.991	0.960	0.947	0.941	0.931	0.920	0.911
Coefficient of variation	0.003	0.007	0.005	0.005	0.008	0.008	0.005
Geometric mean	0.989	0.955	0.943	0.938	0.926	0.914	0.907
Lower 95% CI of geo. mean	0.987	0.950	0.939	0.935	0.921	0.909	0.904
Upper 95% CI of geo. mean	0.991	0.960	0.947	0.941	0.931	0.920	0.911
Harmonic mean	0.989	0.955	0.943	0.938	0.926	0.914	0.907
Lower 95% CI of harm. mean	0.987	0.950	0.939	0.935	0.921	0.909	0.904
Upper 95% CI of harm. mean	0.991	0.960	0.947	0.941	0.931	0.919	0.911
Lower 95% CI of quad. mean	0.987	0.950	0.939	0.935	0.921	0.909	0.904
Upper 95% CI of quad. mean	0.991	0.960	0.947	0.941	0.931	0.920	0.911
Skewness	−3.162	−1.119	0.01067	1.605	−0.7778	1.992	0.2301
Kurtosis	10.000	−0.545	4.022	5.273	3.972	6.310	1.699
Sum	9.887	9.550	9.429	9.380	9.257	9.141	9.070

### Appendix A.4. The Selected Best Sets of Features

Based on the proposed feature selection method, bDBERDTO, the best sets of features extracted from the four adopted datasets are presented in Table A19. These best sets of features are used as inputs to the optimized random forest classifier to achieve the best classification results.

**Table A19:** The selected set of features from the four datasets employed in this work.

Dataset	The Selected Best Set of Features
D1 [41]	Number of times pregnant
	Diastolic blood pressure
	Body mass index
	Diabetes pedigree function
	Age
	Class variable
D2 [50]	Pregnancies
	Glucose
	Blood Pressure
	Diabetes Pedigree Function
	Age
	Outcome
D3 [51]	AGE
	Urea
	Cr
	HbA1c
	HDL
	LDL
	VLDL
	CLASS
D4 [52]	Pregnancies
	Glucose
	Blood Pressure
	BMI
	Diabetes Pedigree Function
	Age
	Outcome
